# Supplementary material for: Lifetime socioeconomic circumstances and chronic pain in later adulthood: findings from a British birth cohort study
Source: BMJ Open. 2019 Mar 7;9(3):e024250. doi: 10.1136/bmjopen-2018-024250 (PMC6429846; doi:10.1136/bmjopen-2018-024250)
Supplement: Supplementary data [file bmjopen-2018-024250supp001.pdf]

Supplementary Table 1. Distribution of indicators of socioeconomic position in childhood by sex.

		Women n (%)	Men n (%)	Total n (%)
Father's occupational class	I-II	308 (26.3)	282 (26.0)	590 (26.1)
	IIINM	242 (20.6)	224 (20.7)	466 (20.6)
	IIIM	332 (28.3)	319 (39.4)	651 (28.8)
	IV-V	291 (24.8)	260 (24.0)	551 (24.4)
	Unknown	65	55	120
Home ownership	Owner-occupier	371 (31.0)	330 (29.9)	701 (30.5)
	Tenant	826 (69.0)	773 (70.1)	1599 (69.5)
	Unknown	41	37	78
Lack of amenities	0	629 (51.9)	597 (53.5)	1226 (52.7)
	1	205 (16.9)	192 (17.2)	397 (17.1)
	2	329 (27.2)	279 (25.0)	608 (26.1)
	3	48 (4.0)	47 (4.2)	95 (4.1)
	Unknown	27	25	52
Overcrowding	Not overcrowded	1128 (94.4)	1064 (96.5)	2192 (95.4)
	Overcrowded	67 (5.6)	39 (3.5)	106 (4.6)
	Unknown	43	37	80
Maternal education	Degree	39 (3.6)	45 (4.4)	84 (4.0)
	Diploma	80 (7.3)	57 (5.6)	137 (6.5)
	Secondary	162 (14.8)	175 (17.2)	337 (15.9)
	Primary	817 (74.4)	742 (72.8)	1,559 (73.6)
	Unknown	140	121	261
Paternal education	Degree	110 (10.1)	102 (10.1)	212 (10.1)
	Diploma	68 (6.2)	58 (5.8)	126 (6.0)
	Secondary	173 (15.9)	184 (18.3)	357 (17.0)
	Primary	740 (67.8)	667 (65.9)	1,404 (66.9)
	Unknown	147	132	279

Supplementary Table 2. Distribution of indicators of socioeconomic position in earlier adulthood by sex.

		Women n (%)	Men n (%)	Total n (%)
Educational level (age 26)	Degree or higher	78 (6.5)	190 (17.2)	268 (11.7)
	A level or equivalent or less	1,116 (93.5)	913 (82.8)	2,029 (88.3)
	Unknown	44	37	81
Hard to manage financially (age 43)*	No	977 (78.9)	911 (88.7)	1888 (87.5)
	Yes	154 (12.4)	116 (11.3)	270 (12.5)
	Unknown	107	113	220
Go without necessities (age 43)*	No	1023 (90.1)	943 (91.6)	1966 (90.8)
	Yes	113 (10.0)	87 (8.5)	200 (9.2)
	Unknown	102	110	212
Unable to pay bills (Age 43)*	No	1082 (95.4)	968 (94.4)	2,050 (94.9)
	Yes	52 (4.6)	58 (5.7)	110 (5.1)
	Unknown	105	115	220
Derived financial hardship (age 43)	None	974 (86.4)	906 (88.7)	1,880 (87.4)
	Minimal	75 (6.7)	61 (6.0)	136 (6.3)
	Moderate	48 (4.3)	32 (3.1)	80 (3.7)
	Most	31 (2.8)	23 (2.3)	54 (2.5)
	Unknown	110	118	228
Occupational class (age 53)	I-II	437 (38.5)	619 (57.1)	1056 (47.6)
	IIINM	418 (36.9)	109 (10.1)	527 (23.8)
	IIIM	86 (7.6)	264 (24.3)	350 (15.8)
	IV-V	193 (17.0)	93 (8.6)	286 (12.9)
	Unknown	104	55	159
Home ownership (age 53)	Owner-occupier	1071 (89.3)	979 (89.4)	2055 (89.4)
	Tenant	128 (10.7)	116 (10.6)	244 (10.6)
	Unknown	40	45	85

* These three variables were used to derive the financial hardship variable.

Supplementary Table 3. Distribution of indicators of socioeconomic position in later adulthood by sex.

		Women n (%)	Men n (%)	Total n (%)
Home ownership	Owner-occupier	802 (73.9)	646 (67.8)	1,448 (71.1)
	Tenant	283 (26.1)	307 (32.2)	590 (29.0)
	Unknown	187	153	340
Hard to manage financially*	No	945 (94.1)	828 (93.1)	1773 (93.7)
	Yes	59 (5.9)	61 (6.9)	120 (6.3)
	Unknown	234	251	485
Go without necessities*	No	911 (90.7)	794 (89.1)	1705 (90.0)
	Yes	93 (9.3)	97 (10.9)	190 (10.0)
	Unknown	234	249	483
Unable to pay bills*	No	974 (97.1)	854 (75.9)	1,828 (96.5)
	Yes	29 (2.9)	37 (4.2)	66 (3.5)
	Unknown	235	249	485
Derived financial hardship	None	944 (94.1)	828 (93.1)	1,772 (93.7)
	Minimal	18 (1.8)	19 (2.1)	37 (2.0)
	Moderate	30 (3.0)	23 (2.6)	53 (2.8)
	Most	11 (1.1)	19 (2.1)	30 (1.6)
	Unknown	237	252	489

* These three variables were used to derive the financial hardship variable.

Supplementary Table 4. Associations between indicators of socioeconomic position in childhood and pain at age 68.

Exposure	n	Model	Exposure level	CWP vs no pain RRR (95% CI)	CRP vs no pain RRR (95% CI)	Other pain vs no pain RRR (95% CI)	p
Father's occupational class (ref = I-II)	2258	1	IIINM	1.11 (0.73, 1.68)	0.94 (0.71, 1.25)	0.90 (0.62, 1.31)	.86
			IIIM	1.05 (0.71, 1.55)	1.09 (0.84, 1.41)	0.87 (0.62, 1.24)	
			IV – V	1.30 (0.87, 1.92)	1.00 (0.76, 1.32)	1.00 (0.70, 1.42)	
		2	IIINM	1.11 (0.73, 1.69)	0.94 (0.71, 1.26)	0.90 (0.62, 1.31)	.87
			IIIM	1.06 (0.71, 1.57)	1.09 (0.84, 1.41)	0.87 (0.62, 1.24)	
			IV – V	1.29 (0.87, 1.92)	1.00 (0.76, 1.32)	1.00 (0.70, 1.42)	
		3	IIINM	1.10 (0.72, 1.67)	0.94 (0.70, 1.25)	0.90 (0.62, 1.31)	.91
			IIIM	0.90 (0.60, 1.34)	1.02 (0.78, 1.33)	0.83 (0.58, 1.18)	
			IV – V	1.07 (0.71, 1.61)	0.94 (0.71, 1.24)	0.96 (0.67, 1.38)	
		4	IIINM	1.10 (0.72, 1.69)	0.93 (0.70, 1.25)	0.90 (0.62, 1.31)	.89
			IIIM	1.02 (0.69, 1.52)	1.07 (0.83, 1.39)	0.87 (0.61, 1.23)	
			IV – V	1.26 (0.84, 1.88)	0.99 (0.75, 1.30)	0.99 (0.70, 1.41)	
		5	IIINM	1.05 (0.68, 1.62)	0.93 (0.70, 1.24)	0.90 (0.62, 1.32)	.91
			IIIM	0.86 (0.57, 1.29)	1.01 (0.77, 1.31)	0.83 (0.58, 1.19)	
			IV – V	1.05 (0.69, 1.59)	0.93 (0.70, 1.23)	0.98 (0.68, 1.41)	
Home ownership (ref = owner)	2300	1	Tenant	1.05 (0.77, 1.42)	1.05 (0.85, 1.29)	1.02 (0.77, 1.34)	.97
		2	Tenant	1.06 (0.78, 1.43)	1.05 (0.85, 1.29)	1.02 (0.77, 1.34)	.96
		3	Tenant	0.92 (0.67, 1.26)	0.98 (0.80, 1.22)	0.98 (0.74, 1.30)	.96
		4	Tenant	1.01 (0.75, 1.38)	1.03 (0.84, 1.27)	.01 (0.77, 1.33)	.99
		5	Tenant	0.88 (0.64, 1.21)	0.97 (0.78, 1.20)	0.98 (0.74, 1.30)	.88
Lack of amenities (ref = lacks 0)	2326	1	Lacks 1 or more	1.22 (0.93, 1.62)	1.03 (0.85, 1.25)	0.99 (0.77, 1.27)	.53
		2	Lacks 1 or more	1.21 (0.92, 1.60)	1.03 (0.85, 1.24)	0.99 (0.77, 1.27)	.57
		3	Lacks 1 or more	1.12 (0.84, 1.48)	0.99 (0.82, 1.21)	0.97 (0.75, 1.25)	.85
		4	Lacks 1 or more	1.24 (0.94, 1.65)	1.04 (0.86, 1.26)	0.99 (0.77, 1.27)	.48
		5	Lacks 1 or more	1.14 (0.85, 1.52)	1.01 (0.83, 1.22)	0.98 (0.76, 1.26)	.81
Overcrowding (ref = not overcrowded)	2298	1	Overcrowded	1.20 (0.64, 2.27)	1.20 (0.77, 1.87)	0.72 (0.36, 1.44)	.47
		2	Overcrowded	1.12 (0.59, 2.11)	1.17 (0.75, 1.82)	0.72 (0.36, 1.44)	.56
		3	Overcrowded	1.06 (0.56, 2.03)	1.15 (0.73, 1.80)	0.70 (0.35, 1.42)	.58
		4	Overcrowded	1.06 (0.56, 2.03)	1.14 (0.73, 1.79)	0.71 (0.35, 1.43)	.60
		5	Overcrowded	1.01 (0.52, 1.95)	1.13 (0.72, 1.77)	0.71 (0.35, 1.44)	.64
Maternal education (ref = dip / degree)	2117	1	Secondary	1.02 (0.56, 1.87)	0.74 (0.50, 1.11)	0.57 (0.34, 0.96)	.42
			Primary	1.07 (0.64, 1.79)	0.81 (0.58, 1.13)	0.75 (0.50, 1.13)	
		2	Secondary	1.06 (0.58, 1.95)	0.76 (0.51, 1.13)	0.56 (0.34, 0.95)	.40
			Primary	1.08 (0.65, 1.82)	0.82 (0.59, 1.13)	0.75 (0.49, 1.13)	
		3	Secondary	1.04 (0.56, 1.91)	0.74 (0.50, 1.10)	0.57 (0.34, 0.96)	.34
			Primary	0.94 (0.55, 1.58)	0.75 (0.53, 1.04)	0.72 (0.48, 1.10)	

		4	Secondary	1.07 (0.58, 1.97)	0.76 (0.51, 1.13)	0.57 (0.34, 0.95)	.40
			Primary	1.11 (0.66, 1.88)	0.82 (0.59, 1.15)	0.75 (0.50, 1.13)	
		5	Secondary	1.01 (0.54, 1.88)	0.74 (0.50, 1.11)	0.57 (0.34, 0.97)	.38
			Primary	0.94 (0.55, 1.61)	0.76 (0.54, 1.06)	0.73 (0.48, 1.11)	
Paternal Education (ref = dip / degree)	2099	1	Secondary	1.08 (0.64, 1.81)	1.24 (0.87, 1.76)	0.83 (0.53, 1.30)	.71
			Primary	1.10 (0.73, 1.66)	1.07 (0.81, 1.42)	0.84 (0.60, 1.20)	
		2	Secondary	1.12 (0.66, 1.87)	1.26 (0.89, 1.78)	0.83 (0.53, 1.30)	.68
			Primary	1.11 (0.73, 1.67)	1.07 (0.81, 1.42)	0.84 (0.60, 1.20)	
		3	Secondary	1.03 (0.61, 1.75)	1.21 (0.85, 1.72)	0.81 (0.51, 1.27)	.60
			Primary	0.89 (0.58, 1.37)	0.97 (0.73, 1.30)	0.80 (0.56, 1.15)	
		4	Secondary	1.09 (0.64, 1.84)	1.24 (0.87, 1.77)	0.82 (0.52, 1.30)	.70
			Primary	1.09 (0.72, 1.66)	1.06 (0.80, 1.41)	0.84 (0.59, 1.19)	
		5	Secondary	0.99 (0.58, 1.69)	1.19 (0.84, 1.70)	0.80 (0.51, 1.27)	.64
			Primary	0.90 (0.58, 1.39)	0.97 (0.72, 1.30)	0.81 (0.57, 1.16)	

1. Unadjusted; 2. Adjusted for sex; 3. Adjusted for sex, BMI, smoking, alcohol, exercise; 4. Adjusted for sex and GHQ-28 caseness; 5. Adjusted for sex, BMI, smoking, alcohol, leisure-time physical activity, GHQ-28 caseness and marital status

Supplementary Table 5. Associations between indicators of socioeconomic position in earlier adulthood and pain at age 68.

Exposure	n	Model	Exposure level	CWP vs no pain<br>RRR (95% CI)	CRP vs no pain<br>RRR (95% CI)	Other pain vs no pain<br>RRR (95% CI)	p
Educational level* Women (ref = degree)	1194	1	A level or equivalent or less	1.10 (0.53, 2.27)	2.04 (1.08, 3.84)	0.54 (0.30, 0.98)	<.01
		3	A level or equivalent or less	0.85 (0.40, 1.79)	1.83 (0.96, 3.46)	0.52 (0.28, 0.96)	<.01
		4	A level or equivalent or less	1.22 (0.58, 2.57)	2.15 (1.14, 4.08)	0.56 (0.31, 1.03)	<.01
		5	A level or equivalent or less	0.94 (0.44, 2.01)	1.93 (1.01, 3.69)	0.54 (0.29, 1.00)	<.01
Educational level* Men (ref = degree)	1103	1	A level or equivalent or less	3.32 (1.41, 7.80)	1.11 (0.77, 1.59)	1.51 (0.93, 2.46)	<.01
		3	A level or equivalent or less	2.64 (1.11, 6.32)	1.06 (0.73, 1.53)	1.45 (0.88, 2.39)	.06
		4	A level or equivalent or less	3.35 (1.41, 7.93)	1.11 (0.78, 1.60)	1.51 (0.93, 2.47)	<.01
		5	A level or equivalent or less	2.68 (1.12, 6.45)	1.07 (0.74, 1.56)	1.45 (0.88, 2.40)	.06
Financial hardship (ref = none)	2150	1	Minimal	1.40 (0.80, 2.46)	1.14 (0.76, 1.71)	0.98 (0.56, 1.71)	<.01
			Moderate	2.73 (1.45, 5.15)	1.34 (0.78, 2.33)	1.31 (0.64, 2.67)	
			Most	5.46 (2.62, 11.39)	1.83 (0.89, 3.74)	1.67 (0.67, 4.18)	
		2	Minimal	1.37 (0.78, 2.42)	1.13 (0.75, 1.70)	0.98 (0.56, 1.71)	<.01
			Moderate	2.61 (1.38, 4.95)	1.31 (0.76, 2.28)	1.31 (0.64, 2.67)	
			Most	5.38 (2.56, 11.30)	1.81 (0.89, 3.72)	1.67 (0.67, 4.18)	
		3	Minimal	1.29 (0.72, 2.28)	1.09 (0.72, 1.64)	0.95 (0.54, 1.66)	<.01
			Moderate	2.49 (1.30, 4.76)	1.33 (0.76, 2.31)	1.32 (0.65, 2.70)	
			Most	4.85 (2.28, 10.35)	1.74 (0.84, 3.58)	1.68 (0.67, 4.23)	
		4	Minimal	1.30 (0.73, 2.30)	1.10 (0.73, 1.66)	0.97 (0.55, 1.69)	<.01
			Moderate	2.44 (1.27, 4.67)	1.27 (0.73, 2.21)	1.29 (0.63, 2.63)	
			Most	4.96 (2.33, 10.57)	1.75 (0.85, 3.60)	1.65 (0.66, 4.13)	
		5	Minimal	1.21 (0.67, 2.17)	1.09 (0.72, 1.65)	0.95 (0.54, 1.68)	.03
			Moderate	2.32 (1.19, 4.52)	1.32 (0.75, 2.30)	1.34 (0.65, 2.74)	
			Most	4.44 (2.02, 9.77)	1.79 (0.86, 3.74)	1.75 (0.69, 4.47)	
Occupational class (ref = I-IIINM)	2219	1	IIINM	1.46 (1.03, 2.09)	1.06 (0.83, 1.36)	1.13 (0.83, 1.54)	0.09
			IIIM	1.25 (0.81, 1.92)	1.28 (0.97, 1.69)	0.98 (0.67, 1.43)	
			IV – V	1.80 (1.17, 2.75)	1.34 (0.99, 1.82)	0.92 (0.60, 1.42)	
		2	IIINM	1.15 (0.79, 1.66)	0.95 (0.73, 1.23)	1.16 (0.83, 1.61)	.15
			IIIM	1.41 (0.92, 2.19)	1.35 (1.02, 1.79)	0.97 (0.66, 1.41)	
			IV – V	1.52 (0.99, 2.34)	1.24 (0.91, 1.69)	0.94 (0.61, 1.45)	
		3	IIINM	1.09 (0.75, 1.59)	0.93 (0.71, 1.20)	1.14 (0.82, 1.59)	.44
			IIIM	1.22 (0.78, 1.90)	1.29 (0.96, 1.71)	0.94 (0.64, 1.39)	
			IV – V	1.26 (0.80, 1.97)	1.19 (0.87, 1.64)	0.93 (0.60, 1.44)	
		4	IIINM	1.11 (0.76, 1.62)	0.93 (0.71, 1.21)	1.15 (0.83, 1.60)	.17
			IIIM	1.45 (0.93, 2.25)	1.36 (1.02, 1.81)	0.97 (0.66, 1.42)	
			IV – V	1.44 (0.93, 2.23)	1.20 (0.88, 1.65)	0.93 (0.60, 1.43)	

		5	IIINM	1.08 (0.74, 1.59)	0.91 (0.70, 1.19)	1.13 (0.81, 1.58)	.38
			IIIM	1.29 (0.82, 2.03)	1.31 (0.98, 1.75)	0.93 (0.63, 1.38)	
			IV – V	1.22 (0.77, 1.92)	1.17 (0.85, 1.61)	0.93 (0.59, 1.44)	
Home ownership (ref = owner)	2294	1	Tenant	1.81 (1.21, 2.70)	1.07 (0.77, 1.48)	1.33 (0.89, 1.98)	.03
		2	Tenant	1.81 (1.21, 2.72)	1.07 (0.77, 1.48)	1.33 (0.89, 1.98)	.03
		3	Tenant	1.60 (1.06, 2.42)	1.02 (0.73, 1.41)	1.33 (0.89, 1.99)	.10
		4	Tenant	1.79 (1.19, 2.69)	1.06 (0.77, 1.47)	1.33 (0.89, 1.98)	.04
		5	Tenant	1.62 (1.06, 2.49)	1.06 (0.76, 1.48)	1.36 (0.90, 2.05)	.11

1. Unadjusted; 2. Adjusted for sex; 3. Adjusted for sex, BMI, smoking, alcohol and leisure-time physical activity; 4. Adjusted for sex and GHQ-28 caseness; 5. Adjusted for sex, BMI, smoking, alcohol, leisure-time physical activity, GHQ-28 caseness and marital status * The models for education are stratified by sex due to a significant interaction term ($p < 0.001$). Model 2 is therefore omitted and no adjustment for sex is made.

Supplementary Table 6. Associations between indicators of socioeconomic position in later adulthood and pain at age 68.

Exposure	n	Model	Exposure level	CWP vs no pain RRR (95% CI)	CRP v no pain RRR (95% CI)	Other pain vs no pain RRR (95% CI)	p
Home ownership (ref = owner)	2038	1	Tenant	1.17 (0.85, 1.62)	1.01 (0.81, 1.26)	0.90 (0.67, 1.22)	.62
		2	Tenant	1.25 (0.90, 1.73)	1.04 (0.83, 1.30)	0.90 (0.67, 1.22)	.44
		3	Tenant	1.08 (0.78, 1.52)	0.97 (0.77, 1.22)	0.88 (0.65, 1.19)	.74
		4	Tenant	1.20 (0.86, 1.67)	1.02 (0.81, 1.28)	0.90 (0.67, 1.21)	.55
		5	Tenant	1.07 (0.76, 1.51)	0.98 (0.78, 1.24)	0.89 (0.66, 1.21)	.83
Financial hardship (ref = none)	1892	1	Minimal	1.55 (0.60, 3.98)	0.87 (0.40, 1.92)	0.77 (0.26, 2.31)	.58
			Moderate	2.06 (0.88, 4.85)	1.74 (0.90, 3.35)	1.54 (0.66, 3.61)	
			Most	1.99 (0.68, 5.80)	1.48 (0.64, 3.43)	0.89 (0.25, 3.22)	
		2	Minimal	1.61 (0.62, 4.16)	0.88 (0.40, 1.94)	0.77 (0.26, 2.30)	.52
			Moderate	2.03 (0.86, 4.80)	1.73 (0.90, 3.34)	1.54 (0.66, 3.62)	
			Most	2.26 (0.77, 6.64)	1.53 (0.66, 3.57)	0.89 (0.24, 3.20)	
		3	Minimal	1.49 (0.57, 3.94)	0.84 (0.38, 1.86)	0.76 (0.25, 2.28)	.64
			Moderate	1.79 (0.74, 4.28)	1.64 (0.85, 3.18)	1.52 (0.64, 3.58)	
			Most	2.35 (0.79, 7.01)	1.51 (0.64, 3.53)	0.89 (0.24, 3.24)	
		4	Minimal	1.48 (0.56, 3.89)	0.84 (0.38, 1.86)	0.75 (0.25, 2.26)	.86
			Moderate	1.61 (0.67, 3.88)	1.54 (0.79, 2.99)	1.46 (0.62, 3.44)	
			Most	1.67 (0.55, 5.04)	1.31 (0.56, 3.09)	0.82 (0.23, 3.00)	
		5	Minimal	1.39 (0.51, 3.74)	0.83 (0.37, 1.87)	0.77 (0.25, 2.34)	.89
			Moderate	1.47 (0.60, 3.63)	1.56 (0.79, 3.05)	1.49 (0.63, 3.54)	
			Most	1.71 (0.55, 5.29)	1.39 (0.58, 3.32)	0.91 (0.25, 3.34)	

1. Unadjusted; 2. Adjusted for sex; 3. Adjusted for sex, BMI, smoking, alcohol and leisure-time physical activity; 4. Adjusted for sex and GHQ-28 caseness; 5. Adjusted for sex, BMI, smoking, alcohol, leisure-time physical activity, GHQ-28 caseness and marital status

Supplementary Table 7. Changes in SEP measures between early and later time periods.

	n (%)	
Tenant status (n = 1,956)	*Tenant status in later adulthood*	
Tenant status in childhood	Owner-occupier	Tenant
Owner-occupier	471 (23.8)	127 (6.4)
Tenant	931 (47.1)	446 (22.6)
Financial hardship (n = 1,795)	*Financial hardship later adulthood*	
Financial hardship earlier adulthood	No (none or minimal)	Yes (moderate or most)
No (none or minimal)	1637 (91.2)	52 (2.9)
Yes (moderate or most)	84 (4.7)	22 (1.2)

Supplementary Table 8. Accumulation of occupational class and financial hardship.

Accumulation	Tenant status	Financial hardship
	n (%)	
0 (high at both time points)	453 (23.2)	1,637 (91.2)
1	1017 (52.0)	136 (7.6)
2	352 (18.0)	22 (1.2)
3	134 (6.9)	-
Unknown	422	583

Supplementary Table 9. Associations between cumulative exposure to tenant status and financial hardship and pain at age 68.

Exposure	n	Model	Exposure level	CWP vs no pain<br>RRR (95% CI)	CRP v no pain<br>RRR (95% CI)	Other pain vs no pain<br>RRR (95% CI)	p
Financial hardship accumulation (ref = No or minimal hardship)	1795	1	Hardship at one point	2.23 (1.32, 3.78)	1.36 (0.88, 2.08)	1.57 (0.93, 2.65)	<.01
			Hardship at both	5.44 (1.80, 16.39)	1.77 (0.59, 5.28)	1.14 (0.23, 5.68)	
		2	Hardship at one point	2.26 (1.33, 3.83)	1.36 (0.89, 2.08)	1.57 (0.93, 2.65)	<.01
			Hardship at both	5.18 (1.70, 15.77)	1.74 (0.58, 5.20)	1.13 (0.23, 5.66)	
		3	Hardship at one point	2.17 (1.27, 3.71)	1.35 (0.88, 2.08)	1.59 (0.93, 2.70)	.02
			Hardship at both	4.45 (1.42, 13.94)	1.63 (0.54, 4.92)	1.12 (0.22, 5.64)	
		4	Hardship at one point	1.99 (1.16, 3.41)	1.28 (0.83, 1.97)	1.53 (0.90, 2.59)	.05
			Hardship at both	4.26 (1.36, 13.32)	1.57 (0.52, 4.76)	1.09 (0.22, 5.45)	
		5	Hardship at one point	1.93 (1.11, 3.35)	1.34 (0.86, 2.08)	1.59 (0.93, 2.73)	.09
			Hardship at both	3.90 (1.20, 12.64)	1.60 (0.52, 4.92)	1.15 (0.23, 5.84)	
Housing ownership accumulation (ref = always owner)	1956	1	Tenant at one point	0.90 (0.61, 1.32)	0.91 (0.70, 1.18)	0.89 (0.63, 1.25)	.47
			Tenant at two points	1.02 (0.63, 1.65)	1.05 (0.76, 1.45)	0.95 (0.62, 1.47)	
			Tenant at all points	1.96 (1.09, 3.52)	1.13 (0.71, 1.80)	1.16 (0.64, 2.11)	
		2	Tenant at one point	0.90 (0.61, 1.33)	0.91 (0.70, 1.18)	0.89 (0.63, 1.25)	.43
			Tenant at two points	1.10 (0.68, 1.79)	1.08 (0.78, 1.50)	0.96 (0.62, 1.47)	
			Tenant at all points	1.99 (1.10, 3.59)	1.13 (0.71, 1.81)	1.17 (0.64, 2.11)	
		3	Tenant at one point	0.84 (0.56, 1.24)	0.86 (0.66, 1.12)	0.86 (0.61, 1.21)	.73
			Tenant at two points	0.93 (0.56, 1.53)	0.98 (0.70, 1.37)	0.90 (0.58, 1.39)	
			Tenant at all points	1.50 (0.81, 2.78)	0.97 (0.60, 1.57)	1.07 (0.58, 1.98)	
		4	Tenant at one point	0.89 (0.60, 1.32)	0.91 (0.70, 1.18)	0.89 (0.63, 1.25)	.60
			Tenant at two points	1.05 (0.64, 1.72)	1.06 (0.76, 1.47)	0.95 (0.62, 1.46)	
			Tenant at all points	1.83 (1.00, 3.34)	1.10 (0.69, 1.76)	1.15 (0.64, 2.09)	
		5	Tenant at one point	0.83 (0.55, 1.23)	0.85 (0.65, 1.11)	0.86 (0.61, 1.21)	.72
			Tenant at two points	0.90 (0.54, 1.49)	0.99 (0.71, 1.39)	0.92 (0.59, 1.43)	
			Tenant at all points	1.48 (0.78, 2.78)	0.99 (0.61, 1.60)	1.12 (0.60, 2.08)	

1. Unadjusted; 2. Adjusted for sex; 3. Adjusted for sex, BMI, smoking, alcohol and leisure-time physical activity; 4. Adjusted for sex and GHQ-28 caseness; 5. Adjusted for sex, BMI, smoking, alcohol, leisure-time physical activity, GHQ-28 caseness and marital status
