# Supplementary material for: Lifetime socioeconomic circumstances and chronic pain in later adulthood: findings from a British birth cohort study
Source: BMJ Open. 2019 Mar 7;9(3):e024250. doi: 10.1136/bmjopen-2018-024250 (PMC6429846; doi:10.1136/bmjopen-2018-024250)
Supplement: Author's manuscript [file bmjopen-2018-024250.draft_revisions.pdf]

BMJ Open

BMJ Open is committed to open peer review. As part of this commitment we make the peer review history of every article we publish publicly available.

When an article is published we post the peer reviewers' comments and the authors' responses online. We also post the versions of the paper that were used during peer review. These are the versions that the peer review comments apply to.

The versions of the paper that follow are the versions that were submitted during the peer review process. They are not the versions of record or the final published versions. They should not be cited or distributed as the published version of this manuscript.

BMJ Open is an open access journal and the full, final, typeset and author-corrected version of record of the manuscript is available on our site with no access controls, subscription charges or pay-per-view fees (http://bmjopen.bmj.com).

If you have any questions on BMJ Open's open peer review process please email info.bmjopen@bmj.com

BMJ Open

Lifetime socioeconomic circumstances and chronic pain in later adulthood: findings from a British birth cohort study

Journal:	*BMJ Open*
Manuscript ID	bmjopen-2018-024250
Article Type:	Research
Date Submitted by the Author:	17-May-2018
Complete List of Authors:	Jay, Matthew; University College London, GOS Institute of Child Health; Great Ormond Street Hospital For Children NHS Foundation Trust, Department of Anaesthesia and Pain Medicine<br>Bendayan, Rebecca; University College London, GOS Institute of Child Health; University College London, MRC Unit for Lifelong Health and Ageing at UCL<br>Cooper, Rachel; University College London, MRC Unit for Lifelong Health and Ageing<br>Muthuri, Stella; MRC Unit for Lifelong Health and Ageing at UCL
Keywords:	EPIDEMIOLOGY, PAIN MANAGEMENT, PUBLIC HEALTH

SCHOLARONE™
Manuscripts

For peer review only

Lifetime socioeconomic circumstances and chronic pain in later adulthood: findings from a British birth cohort study

Matthew A Jay[1,2*]

Rebecca Bendayan[1,3]

Rachel Cooper[3]

Stella Muthuri[3]

1: UCL Great Ormond Street Institute of Child Health: Population, Policy & Practice Programme

2: Great Ormond Street Hospital for Children NHS Foundation Trust: Department of Anaesthesia and Pain Medicine

3: MRC Unit for Lifelong Health and Ageing at UCL

*Corresponding author: matthew.jay.15@ucl.ac.uk

UCL GOS Institute of Child Health

30 Guilford Street

London WC1N 1DT

+44 (0)20 3549 5030

For peer review only - http://bmjopen.bmj.com/site/about/guidelines.xhtml

CONTRIBUTOR STATEMENT

Matthew A Jay (MAJ) had full access to the data and takes full responsibility for the integrity and accuracy of analysis. MAJ, Stella G Muthuri (SGM) and Rebecca Bendayan (RB) conceived the idea for this study. MAJ, SGM, RB and Rachel Cooper contributed to study design. MAJ analysed the data and drafted the manuscript. All authors contributed critically to the manuscript and provided final approval of the version to be published.

For peer review only

For peer review only - http://bmjopen.bmj.com/site/about/guidelines.xhtml

ABSTRACT

Objectives: To investigate associations between a range of different indicators of socioeconomic position (SEP: occupational class, education, household overcrowding and tenure, and experience of financial hardship) across life and chronic widespread and regional pain (CWP and CRP) at age 68.

Design: Prospective birth cohort.

Setting: England, Scotland and Wales.

Participants: Up to 2378 men and women from the MRC National Survey of Health and Development, who have been followed up since birth in 1946 to age 68.

Primary outcome measures: Based on their self-report of pain at age 68, participants were classified as: CWP (American College of Rheumatology criteria), CRP, other pain (<3 months in duration) or no pain.

Results: At age 68, the prevalence of CWP was 13.3% in women and 7.8% in men and that of CRP was 32.3% and 28.7%, respectively. There was no clear evidence that indicators of SEP in childhood or later adulthood were associated with pain. Having experienced (*vs* not) financial hardship and being a tenant (*vs* owner-occupier) in earlier adulthood were both associated with an increased risk of CWP; for example, moderate hardship RRR_{adj} 2.32 [95% CI 1.19, 4.52]; most hardship RRR_{adj} 4.44 [95% CI 2.02, 9.77]. Accumulation of financial hardship across earlier and later adulthood was also associated with an increased risk of CWP.

Conclusions: Consideration of socioeconomic factors in earlier adulthood may be important when identifying targets for intervention to prevent CWP in later life.

For peer review only - http://bmjopen.bmj.com/site/about/guidelines.xhtml

ARTICLE SUMMARY

Strengths and limitations of this study

- We used data from a sample of older adults in Great Britain who were representative of the general population.
- Studying a wide range of indicators of socioeconomic position (SEP) over a 60 year period provided a unique insight into the associations between SEP and chronic widespread and regional pain at age 68.
- Prospective data collection across life reduced the risk of misclassification bias.
- The study might have been subject to survivor bias meaning that associations would be biased towards the null.

For peer review only

For peer review only - http://bmjopen.bmj.com/site/about/guidelines.xhtml

INTRODUCTION

Chronic widespread and regional pain (CWP and CRP) are leading causes of disability globally[1] particularly in later adulthood.[2] In systematic reviews, the prevalence of chronic pain among adults in the UK was estimated to be 44% (95% CI 38%, 49%)[3] and that of CWP to be 11% (95% CI 9%, 13%).[4] Understanding risk factors for CWP and CRP is therefore important to develop preventive strategies. Although the biopsychosocial model of pain emphasises social as well as biological and psychological aspects of pain,[5] there is limited population-level evidence on the role of social factors, particularly socioeconomic position (SEP).[6]

Few studies have examined associations between SEP and CWP, CRP or a combined measure of chronic pain; they have largely been cross-sectional, tending to treat SEP as a confounder.[7-12] They mostly examine only one or two indicators of SEP despite its multifaceted nature[13-15] with different aspects of SEP potentially related to CWP and CRP in different ways. Few also consider the role of SEP across life. Of those that do,[16-18] two using the 1958 British Birth Cohort found associations between lower SEP (paternal and own occupational class during childhood and earlier adulthood, respectively) and higher risk of CWP at age 44-45.[16 17] One of these[16] found lower occupational class was associated with an increased risk of CRP at specific sites; many of these associations, however, were attenuated after adjustment. A paper[18] using the US Health and Retirement Study found higher education and income were associated with lower risk of chronic pain (i.e. arthritis/rheumatism; back or neck problems or received treatment in the past 12 months; frequent or severe headaches; any other chronic pain) in people aged 18 to 64. The extent to which SEP across life is associated with CWP or CRP in participants beyond midlife remains to be determined. Taking a life course approach, by investigating SEP at different life stages,

For peer review only - http://bmjopen.bmj.com/site/about/guidelines.xhtml

could help identify new opportunities for intervention as accumulation of exposure to social stressors across life may be associated with an increased risk of CWP and CRP at older ages.[19]

There are important gaps in knowledge. Firstly, the multifaceted nature of SEP has been neglected in research into the association between SEP, CWP and CRP. Our first aim was therefore to investigate whether a range of different SEP indicators across childhood and adulthood were associated with CWP, CRP or other pain at age 68. Examining CWP and CRP is important as they may have different underlying aetiologies. Secondly, associations between SEP and CWP or CRP in later adulthood cannot be fully understood without considering how SEP operates across life. We therefore also aimed to test whether observed associations are cumulative. We hypothesised that (1) lower levels of each SEP indicator would be associated with an increased risk of CWP (but not necessarily CRP) in later adulthood and (2) that accumulation of low SEP across life would also be associated with increased risk of CWP.

For peer review only - http://bmjopen.bmj.com/site/about/guidelines.xhtml

METHODS

Sample

Data were drawn from the Medical Research Council National Survey of Health and Development, a nationally representative[20 21] longitudinal study of a sample (n=5,362) of single births during the first week of March 1946 in Great Britain.[22] Data collection occurred approximately every two years during childhood and every five to ten years during adulthood, with 24 data collection rounds up to 2014-2015.[20 22 23] Details on overall participation rates, which were generally high, are available elsewhere.[20 24] Of the 2,942 people in the target sample for the most recent data collection at age 68, in 2014-2015, 2,453 (83.4%) completed a postal questionnaire. No attempt was made to contact the remaining 2,420 study members: 957 (17.8%) had already died, 620 (11.6%) had previously withdrawn from the study, 448 (8.3%) had emigrated and were no longer in contact with the study, and 395 (7.4%) had been untraceable for more than five years. Participants who responded were eligible for the present study if they had provided responses to questions on pain (n = 2,378).

Ethical approval was received for each data collection; in 2014-2015 this was obtained from the Queen Square Ethics Committee (14/LO/1073) and the Scotland A Research Ethics Committee (14/SS/1009) and written informed consent was obtained from study members.

Patient and Public involvement statement

Participants have a lifelong association with NSHD. Over the 70 years of the study, the research team has increasingly involved participants, in line with changing norms about conducting cohort studies, starting at age 16 (in 1962) with the annual dissemination of study findings in birthday cards. Participants receive personal letters whenever they raise queries or provide additional comments, including suggestions for new topics to study. In the last ten

For peer review only - http://bmjopen.bmj.com/site/about/guidelines.xhtml

For peer review only

years, the research team has increased the level of participant involvement through invitations to study events (65th and 70th birthday celebrations), public engagement activities, and focus groups to discuss clinical sub-studies. When piloting new questionnaires and assessments, we recruit patients from GP practices or from the UCLH Patient Public Involvement (PPI) group, and take into account their feedback when designing the mainstage fieldwork.

Ascertainment of pain at age 68

Participants were asked: 'In the *last month*, have you had any ache or pain which has lasted for *one day or longer*? (Please do not include pain occurring only during the course of a feverish illness such as flu) [emphasis in original].' Those who answered yes were then asked whether they had been aware of this pain for at least 3 months and were asked to shade on a 4-view body manikin all the areas where they felt pain. Responses to these questions were used to classify participants as having CWP (defined according to the American College of Rheumatology criteria[25] as pain present for 3 months or longer, both above and below the waist; on both the left and right side of the body; and in the axial skeleton). Those who reported pain of at least three months' duration but did not meet the CWP definition were classified as CRP and those reporting pain of less than three months duration were categorised as having other pain.[26]

Indicators of socioeconomic position

A range of SEP indicators were selected for study *a priori* in order to capture different facets of the SEP construct including psychosocial as well as material aspects.[14 15] These indicators were assessed prospectively across life and for the purposes of these analyses were grouped into three life stages (childhood [age 0 to 15]; earlier adulthood [age 26 to 53] and later adulthood [age 60 to 64]). Indicators of childhood SEP were father's occupational class,

For peer review only - http://bmjopen.bmj.com/site/about/guidelines.xhtml

paternal and maternal education, household overcrowding, housing tenure, and lack of household amenities. Where possible, measures were taken at age 4, except for lack of amenities which was ascertained at age 2. However, if data were missing at age 4, comparable measures at ages 11 or 15, were used. See Table 1 for details of categorisation of each measure.

Indicators of SEP in earlier adulthood (i.e. 26 to 53) were highest educational level attained by age 26, financial hardship at age 43 and own occupational class and home ownership at age 53 (or at 43 or 36 if missing at 53) (see Table 1). Indicators of SEP in later adulthood (age 60-64) were home ownership and financial hardship (see Table 1).

Covariates

Potential confounders were identified from the existing literature.[8 16 27-30] BMI ($kg{\cdot}m^{-2}$) was calculated using measures of height and weight recorded by trained nurses at age 60-64. Cigarette smoking status (current, ex or never), participation in sports or vigorous leisure activities in the previous 4 weeks (inactive, 1-4 times per month or 5 or more times per month) and marital status (never married, married/civil partnership, separated/divorced, widowed) were all self-reported at age 68. Participants were asked how often they consumed alcoholic drinks in the last year and to record the number of alcoholic drinks (beers, wines and spirits) they drank in the last 7 days. Units were summed to create an overall measure of alcohol consumptions in units in the previous week at age 68. Affective symptoms were measured using General Health Questionnaire-28 (GHQ-28)[31] score at age 60-64. Items were scored 0-0-1-1, then summed and caseness was identified based on a cut-off of 5 or more.[31]

For peer review only - http://bmjopen.bmj.com/site/about/guidelines.xhtml

Statistical methods

To examine the associations of each SEP indicator with CWP, CRP and other pain at age 68, a series of multinomial logistic regression models were specified. For each SEP indicator, a crude model was first constructed and adjusted for sex. An interaction term between each SEP indicator and sex was entered and subsequent models were sex-stratified if this term was statistically significant (likelihood ratio test, $p < .05$). Categories of covariates were entered as follows. First, BMI and health behaviours (smoking, physical activity and alcohol consumption) were entered. Secondly, adjustment was made for affective symptoms (GHQ-28 caseness). Finally a fully adjusted model which included all covariates was estimated.

To ascertain whether accumulation of low levels of SEP was associated with CWP, CRP or other pain, we derived ordinal indices that indicated, at each time point, whether the participant was in lower or higher SEP (these scores assume equal weight should be given to SEP at each life stage). We only generated these indices for indicators of SEP that had been found at any life stage to be associated with CWP, CRP or other pain and which had been measured at multiple life stages. These indices were entered into regression models and adjustments were carried out in the same order as above.

Models were fit with maximum likelihood and results are presented as relative risk ratios (RRR) with 95% confidence intervals (CIs). Statistical significance of each SEP indicator overall in each model was assessed using the likelihood ratio test. Where covariates were missing (GHQ-28 score [n = 494, 20.8%]; BMI [n = 472, 19.8%]; alcohol consumption [n = 243, 10.2%]; marital status [n = 45, 1.9%]; and smoking status [n = 22, 0.9%]), these were imputed using multiple imputation with chained equations[32] with 40 imputed datasets. The outcome and exposure variables were included in the imputation model but not themselves

For peer review only - http://bmjopen.bmj.com/site/about/guidelines.xhtml

imputed.[33] Parameter estimates were combined using Rubin's rules. Sensitivity analyses exploring these models with only complete cases were performed.

All data management and analysis was conducted in R[34] with RStudio[35] and the packages *mice*[32] and *nnet*.[36]

For peer review only

For peer review only - http://bmjopen.bmj.com/site/about/guidelines.xhtml

RESULTS

The characteristics of the 2,378 participants included in analyses are presented in Table 2. The prevalence of CWP among women and men was 13.3% and 7.8%, respectively. In women, the prevalence of CRP was 32.3% and other pain 12.5%. In men, these figures were 28.7% and 15.2%, respectively. The number and proportion of individuals in the lowest SEP categories is given in Table 2, with a full break-down by sex in Supplementary Tables 1 to 3.

There was no evidence of associations between any of the indicators of childhood SEP and CWP, CRP or other pain at age 68 (Table 3; models at all stages of adjustment in Supplementary Table 4).

Table 4 (and Supplementary Table 5) shows the regression models for each indicator of SEP in earlier adulthood. There were sex differences in the associations between educational attainment and pain (education-sex interaction, $p<.01$). Females with lower levels of education were more likely to report CRP at age 68 compared to those with a degree or higher (RRR 1.93 [95% CI 1.01, 3.69]) and were less likely to report other pain (RRR 0.54, [0.29, 1.00]). Males with lower education levels were more likely to report CWP than males with a degree or higher (2.68 [95% CI 1.12, 6.45]). Financial hardship in earlier adulthood was associated with higher risk of CWP at age 68; fully-adjusted RRR of CWP was 1.21 (95% CI 0.67, 2.17) for minimal hardship, 2.32 (95% CI 1.19, 4.52) for moderate hardship and 4.44 (95% CI 2.02, 9.77) for most financial hardship, compared with no financial hardship. Similar trends were observed for CRP and other pain but the RRRs were smaller and not statistically significant. In a sex-adjusted model, there was some evidence of association between the lowest occupational classes and an increased risk of CWP (RRR 1.52 [95% CI 0.99, 2.34]) which was attenuated in the fully-adjusted model (RRR 1.22 [95% CI

For peer review only - http://bmjopen.bmj.com/site/about/guidelines.xhtml

0.77, 1.92]). Finally, being a tenant (*vs* being an owner-occupier) was associated with an increased risk of CWP at age 68. In a sex-adjusted model the RRR was 1.81 (95% CI 1.21, 2.72), which was slightly attenuated to 1.62 (1.06, 2.49) after adjustment for all covariates.

Neither indicator of SEP in later adulthood (home ownership and financial hardship) was associated with pain at age 68 (Table 5 and Supplementary Table 6).

As tenant status and financial hardship were the only two variables measured at multiple time points that were associated with CWP or CRP, these two were taken forward into the accumulation analyses. The number and proportion of participants in each group at the earliest and latest time points are cross-tabulated in Supplementary Table 7. Supplementary Table 8 shows the number and proportion of participants at each level of the derived accumulation indices.

Results from multinomial logistic regression models examining the cumulative associations between tenant status, financial hardship and CWP, CRP and other pain are shown in Table 6 (and Supplementary Table 9). These show that accumulation of tenant status over life was not associated with pain at age 68. By contrast, accumulation of financial hardship across adulthood was associated with an increased risk of CWP (hardship at one point reported *vs* no hardship RRR 1.93 [95% CI 1.11, 3.35]; hardship at both points RRR 3.90 [95% CI 1.20, 12.64]).

Sensitivity analyses revealed no major differences to the models fit using imputed data (directions of associations were the same and magnitudes were similar) and are not therefore presented.

For peer review only - http://bmjopen.bmj.com/site/about/guidelines.xhtml
For peer review only

DISCUSSION

We aimed to investigate whether different SEP indicators at different life stages were associated with risk of reporting CWP, CRP or other pain at age 68. Experience of financial hardship and tenant status in earlier adulthood were both associated with an increased risk of CWP at age 68. Lower educational attainment was also associated with an increased risk of CWP in men and an increased risk of CRP in women. There was no evidence of associations between any SEP indicator in childhood or later adulthood and any pain outcome at age 68. Our second aim was to assess whether observed associations were cumulative across different life stages and we found that there was a cumulative association between financial hardship during earlier and later adulthood and increased risk of CWP at age 68.

Our finding of stronger and more consistent associations between indicators of SEP in earlier adulthood and pain than with indicators of SEP in childhood or later adulthood, suggests that earlier adulthood may be a sensitive period. Financial hardship appears to be particularly important. This variable captures both subjective and objective elements of hardship (i.e., feeling unable to manage or being unable to pay bills, or both). There may therefore be a combination of psychosocial and material elements that are associated with an increased risk of CWP. It is also especially interesting that financial hardship in later adulthood was not independently associated with CWP but when combined with the same variable in earlier adulthood, a clear inverse association was observed. This suggests that, despite the lack of strong evidence of an association between financial hardship in later adulthood and CWP at 68, intervening to reduce the burden of hardship in later as well as earlier adulthood may prove beneficial in reducing the prevalence of CWP in older age.

For peer review only - http://bmjopen.bmj.com/site/about/guidelines.xhtml

The interaction between education and sex also requires explanation. An increased risk of CWP or CRP with lower levels of education is in line with our initial hypotheses but it is not clear why lower education is associated with an increased risk of CWP among men but with increased risk of CRP among women.

Although we found evidence that earlier adulthood may be a sensitive period, it is also clear that choice of indicator is important as there was limited evidence of an association between occupational class and CWP or CRP. This is surprising given previous findings from the 1958 British Birth Cohort.[16 17] These previous analyses showed that lower parental occupational class in childhood and at age 42 were associated with an increased risk of CWP at age 45 and that the association between own occupational class at 42 and CWP persisted after adjustment for parental occupational class in childhood (and in the case of Macfarlane et al,[16] adjustment for potential confounders/mediators). Reasons for the discrepancy with our findings may be attributable to differences in study design, particularly the ages at which pain was measured. Additionally, social conditions at the time of birth in each of the cohorts were different. Further, the effects of occupation may act only contemporaneously in the aetiology of CWP, for example through the immediate psychosocial or physical demands of the work.[14 15 37 38]

Goosby[18] found that childhood conditions (i.e. self-reported parental education, receipt of financial assistance and having gone hungry) were associated with chronic pain prevalence in adulthood (ages 25-64) before mutually adjusting for adulthood SEP (income, education and benefit receipt since age 18). The latter were all associated with chronic pain in the expected directions. The fact that receipt of benefits was associated with chronic pain in Goosby's study[18] is consistent with our finding that financial hardship was associated with CWP.

For peer review only - http://bmjopen.bmj.com/site/about/guidelines.xhtml

This study has a number of strengths. The NSHD is largely representative of the population of older adults in Great Britain despite some losses to follow up,[22 24] meaning that results are likely to be generalisable to this generation. We examined a wide range of SEP indictors with data being collected over a 60 year period. The study is subject to very limited (if any) recall bias as data were collected prospectively. There is also therefore limited risk of exposure or outcome misclassification (i.e. participants being incorrectly labelled as lower or higher SEP).

Some limitations are also noted. It was not possible to examine incident pain as no data on CWP or CRP had been ascertained before age 68. As onset of CWP and CRP may occur earlier in life and persist into later adulthood, future work will need to establish the temporality of associations. Although we adjusted for a range of covariates, we only did so at one time point. Changes in these variables may be associated with changes in pain state over time and failing to account for their time-varying nature might have resulted in residual confounding. There might also be survivor bias as lower SEP in childhood was associated with lower survival rates into adulthood in NSHD.[39] If so, this would bias associations towards the null and could partially explain why there was no association between childhood SEP and CWP or CRP at age 68. Finally, results might not be generalizable to younger, more ethnically diverse cohorts so the study should be replicated with subsequent generations, particularly the 1958 British Birth Cohort as participants in that study age, which also contains data on SEP and pain.

These results have important implications for public policy. Given its high prevalence, cost and associated disability, prevention of CWP and CRP should be considered a key public

For peer review only - http://bmjopen.bmj.com/site/about/guidelines.xhtml

health priority. Policy makers should ensure that socioeconomic adversity across life is addressed in public health interventions, particularly the role that housing, education and financial hardship may play.

For peer review only

For peer review only - http://bmjopen.bmj.com/site/about/guidelines.xhtml

ACKNOWLEDGMENTS

The authors are grateful to NSHD study members for their continuing participation in the study. We also thank members of the NSHD scientific and data collection teams.

FUNDING STATEMENT

This work was supported by the UK Medical Research Council which provides core funding for the MRC National Survey of Health and Development and supported SGM and RC (programme code: MC_UU_12019/4). SGM was also supported by MRC grant MR/L010399/1. MAJ was supported while carrying out this work by a grant from Great Ormond Street Hospital for Children NHS Foundation Trust.

COMPETING INTERESTS

The authors have no conflicts of interest to declare.

DATA SHARING STATEMENT

Data used in this publication are available to bona fide researchers upon request to the NSHD Data Sharing Committee via a standard application procedure. Further details can be found at http://www.nshd.mrc.ac.uk/data. doi: 10.5522/NSHD/Q101; doi: 10.5522/NSHD/Q102; 10.5522/NSHD/Q103.

For peer review only - http://bmjopen.bmj.com/site/about/guidelines.xhtml

REFERENCES

1. Rice AS, Smith BH, Blyth FM. Pain and the global burden of disease. *Pain* 2016;157(4):791-6.

2. Thomas E. Pain in older people. In: Croft P, Blyth FM, van Der Windt D, eds. Chronic Pain Epidemiology: From Aetiology to Public Health. Oxford: OUP 2010:185-99.

3. Fayaz A, Croft P, Langford RM, et al. Prevalence of chronic pain in the UK: a systematic review and meta-analysis of population studies. *BMJ Open* 2016;6(6).

4. Mansfield KE, Sim J, Jordan JL, et al. A systematic review and meta-analysis of the prevalence of chronic widespread pain in the general population. *Pain* 2016;157(1):55-64.

5. Gatchel RJ, Peng YB, Peters ML, et al. The biopsychosocial approach to chronic pain: scientific advances and future directions. *Psychol Bull* 2007;133(4):581.

6. Blyth FM, Macfarlane GJ, Nicholas MK. The contribution of psychosocial factors to the development of chronic pain: the key to better outcomes for patients? *Pain* 2007;129(1-2):8-11.

7. Blyth FM, March LM, Brnabic AJ, et al. Chronic pain in Australia: a prevalence study. *Pain* 2001;89(2-3):127-34.

8. Bridges S. Chapter 9 Chronic Pain. In: Craig R, Mindell J, eds. Health Survey for England 2011. Leeds: Health and Social Care Information Centre 2011.

9. Elliott AM, Smith BH, Penny KI, et al. The epidemiology of chronic pain in the community. *Lancet* 1999;354(9186):1248-52.

10. Hagen K, Zwart JA, Svebak S, et al. Low socioeconomic status is associated with chronic musculoskeletal complaints among 46,901 adults in Norway. *Scand J Public Health* 2005;33(4):268-75.

For peer review only - http://bmjopen.bmj.com/site/about/guidelines.xhtml

11. Raftery MN, Sarma K, Murphy AW, et al. Chronic pain in the Republic of Ireland--community prevalence, psychosocial profile and predictors of pain-related disability: results from the Prevalence, Impact and Cost of Chronic Pain (PRIME) study, part 1. *Pain* 2011;152(5):1096-103.

12. Toblin RL, Mack KA, Perveen G, et al. A population-based survey of chronic pain and its treatment with prescription drugs. *Pain* 2011;152(6):1249-55.

13. Galobardes B, Lynch J, Smith GD. Measuring socioeconomic position in health research. *Br Med Bull* 2007;81-82:21-37.

14. Galobardes B, Shaw M, Lawlor DA, et al. Indicators of socioeconomic position (part 1). *J Epidemiol Comm Health* 2006;60(1):7-12.

15. Galobardes B, Shaw M, Lawlor DA, et al. Indicators of socioeconomic position (part 2). *J Epidemiol Comm Health* 2006;60(2):95-101.

16. Macfarlane GJ, Norrie G, Atherton K, et al. The influence of socioeconomic status on the reporting of regional and widespread musculoskeletal pain: results from the 1958 British Birth Cohort Study. *Ann Rheum Dis* 2009;68(10):1591-5.

17. Power C, Atherton K, Strachan DP, et al. Life-course influences on health in British adults: effects of socio-economic position in childhood and adulthood. *Int J Epidemiol* 2007;36(3):532-39.

18. Goosby BJ. Early life course pathways of adult depression and chronic pain. *J Health Soc Behav* 2013;54(1):75-91.

19. Ben-Shlomo Y, Kuh D. A life course approach to chronic disease epidemiology: conceptual models, empirical challenges and interdisciplinary perspectives. *Int J Epidemiol* 2002;31(2):285-93.

For peer review only - http://bmjopen.bmj.com/site/about/guidelines.xhtml

20. Wadsworth M, Kuh D, Richards M, et al. Cohort Profile: The 1946 National Birth Cohort (MRC National Survey of Health and Development). *Int J Epidemiol* 2006;35(1):49-54.

21. Wadsworth ME, Butterworth SL, Hardy RJ, et al. The life course prospective design: an example of benefits and problems associated with study longevity. *Soc Sci Med* 2003;57(11):2193-205.

22. Stafford M, Black S, Shah I, et al. Using a birth cohort to study ageing: representativeness and response rates in the National Survey of Health and Development. *Eur J Ageing* 2013;10(2):145-57.

23. Kuh D, Pierce M, Adams J, et al. Cohort profile: updating the cohort profile for the MRC National Survey of Health and Development: a new clinic-based data collection for ageing research. *Int J Epidemiol* 2011;40(1):e1-9.

24. Kuh D, Wong A, Shah I, et al. The MRC National Survey of Health and Development reaches age 70: maintaining participation at older ages in a birth cohort study. *Eur J Epidemiol* 2016;31(11):1135-47.

25. Wolfe F, Smythe HA, Yunus MB, et al. The American College of Rheumatology 1990 Criteria for the Classification of Fibromyalgia. Report of the Multicenter Criteria Committee. *Arthritis Rheum* 1990;33(2):160-72.

26. Muthuri SG, Kuh D, Bendayan R, et al. Chronic physical illness in early life and risk of chronic widespread and regional pain at age 68: evidence from the 1946 British birth cohort. *Pain* 2016;157(10):2382-9.

27. Heim N, Snijder MB, Deeg DJ, et al. Obesity in older adults is associated with an increased prevalence and incidence of pain. *Obesity* 2008;16(11):2510-7.

For peer review only - http://bmjopen.bmj.com/site/about/guidelines.xhtml

28. Mundal I, Grawe RW, Bjorngaard JH, et al. Prevalence and long-term predictors of persistent chronic widespread pain in the general population in an 11-year prospective study: the HUNT study. *BMC Musculoskelet Disord* 2014;15:213

29. Hartvigsen J, Christensen K. Active lifestyle protects against incident low back pain in seniors: a population-based 2-year prospective study of 1387 Danish twins aged 70-100 years. *Spine* 2007;32(1):76-81.

30. Elliott AM, Smith BH, Hannaford PC, et al. The course of chronic pain in the community: results of a 4-year follow-up study. *Pain* 2002;99(1-2):299-307.

31. Goldberg DP, Hillier VF. A scaled version of the General Health Questionnaire. *Psychol Med* 1979;9(1):139-45.

32. van Buuren S, Groothuis-Oudshoorn K. mice: Multivariate Imputation by Chained Equations in R. *J Stat Soft* 2011;45(3):67.

33. Sterne JAC, White IR, Carlin JB, et al. Multiple imputation for missing data in epidemiological and clinical research: potential and pitfalls. *BMJ* 2009;338:b2393.

34. R: A language and environment for statistical computing [program]. Vienna, Austria: R Foundation for Statistical Computing, 2015.

35. RStudio: Integrated Development for R [program]. Boston, MA, USA: RStudio, Inc, 2015.

36. Modern Applied Statistics with S [program]. New York: Springer, 2002.

37. Woods V. Work-related musculoskeletal health and social support. *Occupational Med (Oxford)* 2005;55(3):177-89.

38. Christensen JO, Knardahl S. Work and neck pain: a prospective study of psychological, social, and mechanical risk factors. *Pain* 2010;151(1):162-73.

For peer review only

For peer review only - http://bmjopen.bmj.com/site/about/guidelines.xhtml

39. Kuh D, Hardy R, Langenberg C, et al. Mortality in adults aged 26-54 years related to socioeconomic conditions in childhood and adulthood: post war birth cohort study. *BMJ* 2002;325(7372):1076-80.

For peer review only

For peer review only - http://bmjopen.bmj.com/site/about/guidelines.xhtml

Table 1. Indicators of socioeconomic position used in analyses

Indicator	Definition	Age ascertained (ages used if missing at main age)
Childhood		
Paternal occupational class	Registrar General's Social Classification:<br>I & II – Professional & Managerial and technical<br>IIINM – Skilled non-manual<br>IIIM – Skilled manual<br>IV & V – Partly-skilled & Unskilled	4 (or 11 (n = 48) or 15 (n = 17))<br>Total = 2,258
Paternal and maternal education	Higher<br>Secondary only<br>Primary / less	6 (Paternal = 2,117; Maternal = 2,099)
Household overcrowding	> 2 persons per room in the household	4 (11 (n = 34) 15 (n = 13))<br>Total = 2,298
Housing tenure	Owner-occupier vs<br>Tenant (includes private and public tenants)	4 (or 11 (n = 42) or 15 (n = 12))<br>Total = 2,300
Lack of household amenities	Lacks access to hot running water, own bathroom or own kitchen (score indicating lacks 0 to all 3).	2 (or 11 (n = 157) or 15 (n = 11))<br>Total = 2,326
Earlier adulthood		
Own occupational class	Registrar General's Social Classification (categorised as above)	53 (or 43 (n = 123) or 36 (n = 59))<br>Total = 2,219
Home ownership	Owner-occupier vs<br>Tenant (includes private and public tenants)	53 (or 43 (n = 142) or 36 (n = 29))<br>Total = 2,294
Highest educational level attained	Degree or higher<br>A level or equivalent<br>O level or equivalent<br>None	26 (n = 2,297)
Financial hardship	None (no hardship)<br>Minimal (hard to manage but does not report going without necessities or unable to pay bills)<br>Moderate (hard to manage and either went without necessities or unable to pay bills)<br>Most (hard to manage, went without necessities and unable to pay bills)	43 (n = 2,150)
Later adulthood		
Home ownership	As in earlier adulthood	60-64 (n = 2,038)
Financial hardship	As in earlier adulthood	60-64 (n = 1,892)

For peer review only - http://bmjopen.bmj.com/site/about/guidelines.xhtml

Table 2. Characteristics of participants from of the MRC National Survey of Health and Development included in analyses (sample restricted to those with data on pain at age 68 and at least one indicator of SEP (maximum n=2,378))

		Females (n = 1,140)*	Males (n = 1,238)*
Pain at age 68			
	CWP	164 (13.3%)	89 (7.8%)
	CRP	400 (32.3%)	327 (28.7%)
	Other pain	155 (12.5%)	173 (15.2%)
	No pain	519 (41.9%)	551 (48.3%)
BMI at age 60-64; n = 1,906			
	$kg{\cdot}m^{-2}$ mean (SD)	27.8 (5.2)	27.8 (4.0)
Smoking at age 68, n = 2,356			
	Current	96 (7.8%)	112 (9.9%)
	Ex-smoker	653 (53.2%)	680 (60.2%)
	Never	478 (39.0%)	337 (29.9%)
Alcohol units at age 68, n = 2,135		2.0 (0.0, 6.0)	7.0 (2.0, 15.0)
Leisure time physical activity at age 68, n = 2,352			
	Inactive	733 (59.7%)	682 (60.7%)
	1 to 4 times / month	163 (13.3%)	120 (10.6%)
	5 or more times / month	330 (26.9%)	324 (28.7%)
Symptoms of anxiety and depression (GHQ-28) at age 60-64, n = 1,884			
	Yes (≤4)	222 (22.4%)	115 (12.9%)
	No (>4)	768 (77.6%)	779 (87.1%)
Marital status at age 68, n = 2,333			
	Single	39 (3.2%)	47 (4.2%)
	Married	856 (70.7%)	912 (81.2%)
	Separated / divorced	167 (13.8%)	109 (9.7%)
	Widowed	148 (12.2%)	55 (4.9%)
Childhood SEP (age 0 – 15)			
Father's occupational class	Class IV to V	291 (24.8%)	260 (24.0%)
Home ownership	Tenant status	826 (69.0%)	773 (70.1%)
Lack of amenities	Lacks three amenities	48 (4.0%)	47 (4.2%)
Overcrowding	Overcrowded	67 (5.6%)	39 (3.5%)
Maternal education	Primary or less	817 (74.4%)	742 (72.8%)
Paternal education	Primary or less	740 (67.8%)	667 (65.9%)
Earlier adulthood SEP (age 26 – 53)			
Education	None	390 (32.7%)	378 (34.3%)
Financial hardship	Most hardship	31 (2.8%)	23 (2.3%)
Occupational class	Class IV to V	193 (17.0%)	93 (8.6%)
Home ownership	Tenant status	128 (10.7%)	116 (10.6%)
Later adulthood SEP (age 60/64)			
Home ownership	Tenant status	283 (26.1%)	307 (32.2%)
Financial hardship	Most hardship	11 (1.1%)	19 (2.1%)

For brevity descriptive statistics for only the most adverse category of each SEP indicator are presented, a complete breakdown of the distribution of each SEP indicator by sex is given in Supplementary Tables 1 and 2. Numbers on each covariate vary due to missing data. CWP Chronic widespread pain; CRP Chronic regional pain; GHQ-28 General Health Questionnaire – 28 item; BMI Body Mass Index; SEP socioeconomic position.

For peer review only - http://bmjopen.bmj.com/site/about/guidelines.xhtml

Table 3. Associations between indicators of socioeconomic position in childhood and pain at age 68.

Exposure	n	Model	Exposure level	CWP v no pain RRR (95% CI)	CRP v no pain RRR (95% CI)	Other pain vs no pain RRR (95% CI)	p
Father's occupational class (ref = I-II)	2,258	1	IIINM	1.11 (0.73, 1.69)	0.94 (0.71, 1.26)	0.90 (0.62, 1.31)	.87
			IIIM	1.06 (0.71, 1.57)	1.09 (0.84, 1.41)	0.87 (0.62, 1.24)	
			IV – V	1.29 (0.87, 1.92)	1.00 (0.76, 1.32)	1.00 (0.70, 1.42)	
		2	IIINM	1.05 (0.68, 1.62)	0.93 (0.70, 1.24)	0.90 (0.62, 1.32)	.97
			IIIM	0.86 (0.57, 1.29)	1.01 (0.77, 1.31)	0.83 (0.58, 1.19)	
			IV – V	1.05 (0.69, 1.59)	0.93 (0.70, 1.23)	0.98 (0.68, 1.41)	
Home ownership (ref = owner-occupier)	2,300	1	Tenant	1.06 (0.78, 1.43)	1.05 (0.85, 1.29)	1.02 (0.77, 1.34)	.96
		2	Tenant	0.88 (0.64, 1.21)	0.97 (0.78, 1.20)	0.98 (0.74, 1.30)	.88
Lack of amenities (ref = lacks 0)	2,326	1	Lacks 1 or more	1.21 (0.92, 1.60)	1.03 (0.85, 1.24)	0.99 (0.77, 1.27)	.57
		2	Lacks 1 or more	1.14 (0.85, 1.52)	1.01 (0.83, 1.22)	0.98 (0.76, 1.26)	.81
Overcrowding (ref = not overcrowded)	2,298	1	Overcrowded	1.12 (0.59, 2.11)	1.17 (0.75, 1.82)	0.72 (0.36, 1.44)	.56
		2	Overcrowded	1.01 (0.52, 1.95)	1.13 (0.72, 1.77)	0.71 (0.35, 1.44)	.64
Maternal education (ref = diploma / degree)	2,117	1	Secondary	1.06 (0.58, 1.95)	0.76 (0.51, 1.13)	0.56 (0.34, 0.95)	.40
			Primary	1.08 (0.65, 1.82)	0.82 (0.59, 1.13)	0.75 (0.49, 1.13)	
		2	Secondary	1.01 (0.54, 1.88)	0.74 (0.50, 1.11)	0.57 (0.34, 0.97)	.38
			Primary	0.94 (0.55, 1.61)	0.76 (0.54, 1.06)	0.73 (0.48, 1.11)	
Paternal education (ref = diploma / degree)	2,099	1	Secondary	1.12 (0.66, 1.87)	1.26 (0.89, 1.78)	0.83 (0.53, 1.30)	.68
			Primary	1.11 (0.73, 1.67)	1.07 (0.81, 1.42)	0.84 (0.60, 1.20)	
		2	Secondary	0.99 (0.58, 1.69)	1.19 (0.84, 1.70)	0.80 (0.51, 1.27)	.64
			Primary	0.90 (0.58, 1.39)	0.97 (0.72, 1.30)	0.81 (0.57, 1.16)	

Model 1: Sex-adjusted; Model 2: adjusted for sex, BMI, smoking, alcohol, leisure-time physical activity, GHQ-28 caseness and marital status. CWP Chronic widespread pain; CRP Chronic regional pain.

For peer review only

For peer review only - http://bmjopen.bmj.com/site/about/guidelines.xhtml

Table 4. Associations between indicators of socioeconomic position in earlier and later adulthood and pain at age 68.

Exposure	n	Model	Exposure level	CWP v no pain RRR (95% CI)	CRP v no pain RRR (95% CI)	Other pain vs no pain RRR (95% CI)	p
Earlier adulthood							
Education (females)* (ref = degree)	1,194	2	Secondary or less	0.94 (0.44, 2.01)	1.93 (1.01, 3.69)	0.54 (0.29, 1.00)	<.01
Education (males)* (ref = degree)	1,103	2	Secondary or less	2.68 (1.12, 6.45)	1.07 (0.74, 1.56)	1.45 (0.88, 2.40)	.06
Financial hardship (ref = none)	2,150	1	Minimal	1.37 (0.78, 2.42)	1.13 (0.75, 1.70)	0.98 (0.56, 1.71)	<.01
			Moderate	2.61 (1.38, 4.95)	1.31 (0.76, 2.28)	1.31 (0.64, 2.67)	
			Most	5.38 (2.56, 11.30)	1.81 (0.89, 3.72)	1.67 (0.67, 4.18)	
		2	Minimal	1.21 (0.67, 2.17)	1.09 (0.72, 1.65)	0.95 (0.54, 1.68)	.03
			Moderate	2.32 (1.19, 4.52)	1.32 (0.75, 2.30)	1.34 (0.65, 2.74)	
			Most	4.44 (2.02, 9.77)	1.79 (0.86, 3.74)	1.75 (0.69, 4.47)	
Occupational class (ref = I-II)	2,219	1	IIINM	1.15 (0.79, 1.66)	0.95 (0.73, 1.23)	1.16 (0.83, 1.61)	.15
			IIIM	1.41 (0.92, 2.19)	1.35 (1.02, 1.79)	0.97 (0.66, 1.41)	
			IV – V	1.52 (0.99, 2.34)	1.24 (0.91, 1.69)	0.94 (0.61, 1.45)	
		2	IIINM	1.08 (0.74, 1.59)	0.91 (0.70, 1.19)	1.13 (0.81, 1.58)	.38
			IIIM	1.29 (0.82, 2.03)	1.31 (0.98, 1.75)	0.93 (0.63, 1.38)	
			IV – V	1.22 (0.77, 1.92)	1.17 (0.85, 1.61)	0.93 (0.59, 1.44)	
Home ownership (ref = owner-occupier)	2,294	1	Tenant	1.81 (1.21, 2.72)	1.07 (0.77, 1.48)	1.33 (0.89, 1.98)	.03
		2	Tenant	1.62 (1.06, 2.49)	1.06 (0.76, 1.48)	1.36 (0.90, 2.05)	.11
Later adulthood							
Home ownership (ref = owner)	2,038	1	Tenant	1.25 (0.90, 1.73)	1.04 (0.83, 1.30)	0.90 (0.67, 1.22)	.44
		2	Tenant	1.07 (0.76, 1.51)	0.98 (0.78, 1.24)	0.89 (0.66, 1.21)	.83
Financial hardship (ref = none)	1,892	1	Minimal	1.61 (0.62, 4.16)	0.88 (0.40, 1.94)	0.77 (0.26, 2.30)	.52
			Moderate	2.03 (0.86, 4.80)	1.73 (0.90, 3.34)	1.54 (0.66, 3.62)	
			Most	2.26 (0.77, 6.64)	1.53 (0.66, 3.57)	0.89 (0.24, 3.20)	
		2	Minimal	1.39 (0.51, 3.74)	0.83 (0.37, 1.87)	0.77 (0.25, 2.34)	.89
			Moderate	1.47 (0.60, 3.63)	1.56 (0.79, 3.05)	1.49 (0.63, 3.54)	
			Most	1.71 (0.55, 5.29)	1.39 (0.58, 3.32)	0.91 (0.25, 3.34)	

Model 1: Sex-adjusted; Model 2: adjusted for sex, BMI, smoking, alcohol, leisure-time physical activity, GHQ-28 caseness and marital status.* The models for education are stratified by sex due to a significant interaction term between sex and education (Likelihood ratio test, $p < 0.001$). CWP Chronic widespread pain; CRP Chronic regional pain.

For peer review only - http://bmjopen.bmj.com/site/about/guidelines.xhtml

Table 5. Associations between cumulative exposure to tenant status and financial hardship and pain at age 68.

Exposure	n	Model	Accumulation	CWP vs no pain	CRP vs no pain RRR (95% CI)	Other pain vs no pain RRR (95% CI)	p
Housing ownership accumulation (ref = always owner)	1,956	1	Tenant at one point	0.90 (0.61, 1.33)	0.91 (0.70, 1.18)	0.89 (0.63, 1.25)	.43
			Tenant at two points	1.10 (0.68, 1.79)	1.08 (0.78, 1.50)	0.96 (0.62, 1.47)	
			Tenant at all points	1.99 (1.10, 3.59)	1.13 (0.71, 1.81)	1.17 (0.64, 2.11)	
		2	Tenant at one point	0.83 (0.55, 1.23)	0.85 (0.65, 1.11)	0.86 (0.61, 1.21)	.72
			Tenant at two points	0.90 (0.54, 1.49)	0.99 (0.71, 1.39)	0.92 (0.59, 1.43)	
			Tenant at all points	1.48 (0.78, 2.78)	0.99 (0.61, 1.60)	1.12 (0.60, 2.08)	
Financial hardship accumulation (ref = No or minimal hardship)	1,795	1	Hardship at one point	2.26 (1.33, 3.83)	1.36 (0.89, 2.08)	1.57 (0.93, 2.65)	<.01
			Hardship at both points	5.18 (1.70, 15.77)	1.74 (0.58, 5.20)	1.13 (0.23, 5.66)	
		2	Hardship at one point	1.93 (1.11, 3.35)	1.34 (0.86, 2.08)	1.59 (0.93, 2.73)	.09
			Hardship at both points	3.90 (1.20, 12.64)	1.60 (0.52, 4.92)	1.15 (0.23, 5.84)	

Model 1: Sex-adjusted; Model 2: adjusted for sex, BMI, smoking, alcohol, leisure-time physical activity, GHQ-28 caseness and marital status. CWP Chronic widespread pain; CRP Chronic regional pain.

For peer review only

For peer review only - http://bmjopen.bmj.com/site/about/guidelines.xhtml

Supplementary Table 1. Distribution of indicators of socioeconomic position in childhood by sex.

		Females n (%)	Males n (%)	Total n (%)
Father's occupational class	I-II	308 (26.3)	282 (26.0)	590 (26.1)
	IIINM	242 (20.6)	224 (20.7)	466 (20.6)
	IIIM	332 (28.3)	319 (39.4)	651 (28.8)
	IV-V	291 (24.8)	260 (24.0)	551 (24.4)
	Unknown	65	55	120
Home ownership	Owner-occupier	371 (31.0)	330 (29.9)	701 (30.5)
	Tenant	826 (69.0)	773 (70.1)	1599 (69.5)
	Unknown	41	37	78
Lack of amenities	0	629 (51.9)	597 (53.5)	1226 (52.7)
	1	205 (16.9)	192 (17.2)	397 (17.1)
	2	329 (27.2)	279 (25.0)	608 (26.1)
	3	48 (4.0)	47 (4.2)	95 (4.1)
	Unknown	27	25	52
Overcrowding	Not overcrowded	1128 (94.4)	1064 (96.5)	2192 (95.4)
	Overcrowded	67 (5.6)	39 (3.5)	106 (4.6)
	Unknown	43	37	80
Maternal education	Degree	39 (3.6)	45 (4.4)	84 (4.0)
	Diploma	80 (7.3)	57 (5.6)	137 (6.5)
	Secondary	162 (14.8)	175 (17.2)	337 (15.9)
	Primary	817 (74.4)	742 (72.8)	1,559 (73.6)
	Unknown	140	121	261
Paternal education	Degree	110 (10.1)	102 (10.1)	212 (10.1)
	Diploma	68 (6.2)	58 (5.8)	126 (6.0)
	Secondary	173 (15.9)	184 (18.3)	357 (17.0)
	Primary	740 (67.8)	667 (65.9)	1,404 (66.9)
	Unknown	147	132	279

For peer review only

Supplementary Table 2. Distribution of indicators of socioeconomic position in earlier adulthood by sex.

		Females n (%)	Males n (%)	Total n (%)
Education (age 26)	Degree	78 (6.5)	190 (17.2)	268 (11.7)
	GCE	623 (52.2)	478 (43.3)	1101 (47.9)
	Primary	103 (8.6)	57 (5.2)	160 (7.0)
	None	390 (32.7)	378 (34.3)	768 (33.4)
	Unknown	44	37	81
Hard to manage financially (age 43)*	No	977 (78.9)	911 (88.7)	1888 (87.5)
	Yes	154 (12.4)	116 (11.3)	270 (12.5)
	Unknown	107	113	220
Go without necessities (age 43)*	No	1023 (90.1)	943 (91.6)	1966 (90.8)
	Yes	113 (10.0)	87 (8.5)	200 (9.2)
	Unknown	102	110	212
Unable to pay bills (Age 43)*	No	1082 (95.4)	968 (94.4)	2,050 (94.9)
	Yes	52 (4.6)	58 (5.7)	110 (5.1)
	Unknown	105	115	220
Derived financial hardship (age 43)	None	974 (86.4)	906 (88.7)	1,880 (87.4)
	Minimal	75 (6.7)	61 (6.0)	136 (6.3)
	Moderate	48 (4.3)	32 (3.1)	80 (3.7)
	Most	31 (2.8)	23 (2.3)	54 (2.5)
	Unknown	110	118	228
Occupational class (age 53)	I-II	437 (38.5)	619 (57.1)	1056 (47.6)
	IIINM	418 (36.9)	109 (10.1)	527 (23.8)
	IIIM	86 (7.6)	264 (24.3)	350 (15.8)
	IV-V	193 (17.0)	93 (8.6)	286 (12.9)
	Unknown	104	55	159
Home ownership (age 53)	Owner-occupier	1071 (89.3)	979 (89.4)	2055 (89.4)
	Tenant	128 (10.7)	116 (10.6)	244 (10.6)
	Unknown	40	45	85

* These three variables were used to derive the financial hardship variable.

For peer review only

Supplementary Table 3. Distribution of indicators of socioeconomic position in later adulthood by sex.

		Females n (%)	Males n (%)	Total n (%)
Home ownership	Owner-occupier	802 (73.9)	646 (67.8)	1,448 (71.1)
	Tenant	283 (26.1)	307 (32.2)	590 (29.0)
	Unknown	187	153	340
Hard to manage financially*	No	945 (94.1)	828 (93.1)	1773 (93.7)
	Yes	59 (5.9)	61 (6.9)	120 (6.3)
	Unknown	234	251	485
Go without necessities*	No	911 (90.7)	794 (89.1)	1705 (90.0)
	Yes	93 (9.3)	97 (10.9)	190 (10.0)
	Unknown	234	249	483
Unable to pay bills*	No	974 (97.1)	854 (75.9)	1,828 (96.5)
	Yes	29 (2.9)	37 (4.2)	66 (3.5)
	Unknown	235	249	485
Derived financial hardship	None	944 (94.1)	828 (93.1)	1,772 (93.7)
	Minimal	18 (1.8)	19 (2.1)	37 (2.0)
	Moderate	30 (3.0)	23 (2.6)	53 (2.8)
	Most	11 (1.1)	19 (2.1)	30 (1.6)
	Unknown	237	252	489

* These three variables were used to derive the financial hardship variable.

For peer review only

Supplementary Table 4. Associations between indicators of socioeconomic position in childhood and pain at age 68.

Exposure	n	Model	Exposure level	CWP vs no pain RRR (95% CI)	CRP vs no pain RRR (95% CI)	Other pain vs no pain RRR (95% CI)	p
Father's occupational class (ref = I-II)	2258	1	IIINM	1.11 (0.73, 1.68)	0.94 (0.71, 1.25)	0.90 (0.62, 1.31)	.86
			IIIM	1.05 (0.71, 1.55)	1.09 (0.84, 1.41)	0.87 (0.62, 1.24)	
			IV – V	1.30 (0.87, 1.92)	1.00 (0.76, 1.32)	1.00 (0.70, 1.42)	
		2	IIINM	1.11 (0.73, 1.69)	0.94 (0.71, 1.26)	0.90 (0.62, 1.31)	.87
			IIIM	1.06 (0.71, 1.57)	1.09 (0.84, 1.41)	0.87 (0.62, 1.24)	
			IV – V	1.29 (0.87, 1.92)	1.00 (0.76, 1.32)	1.00 (0.70, 1.42)	
		3	IIINM	1.10 (0.72, 1.67)	0.94 (0.70, 1.25)	0.90 (0.62, 1.31)	.91
			IIIM	0.90 (0.60, 1.34)	1.02 (0.78, 1.33)	0.83 (0.58, 1.18)	
			IV – V	1.07 (0.71, 1.61)	0.94 (0.71, 1.24)	0.96 (0.67, 1.38)	
		4	IIINM	1.10 (0.72, 1.69)	0.93 (0.70, 1.25)	0.90 (0.62, 1.31)	.89
			IIIM	1.02 (0.69, 1.52)	1.07 (0.83, 1.39)	0.87 (0.61, 1.23)	
			IV – V	1.26 (0.84, 1.88)	0.99 (0.75, 1.30)	0.99 (0.70, 1.41)	
		5	IIINM	1.05 (0.68, 1.62)	0.93 (0.70, 1.24)	0.90 (0.62, 1.32)	.91
			IIIM	0.86 (0.57, 1.29)	1.01 (0.77, 1.31)	0.83 (0.58, 1.19)	
			IV – V	1.05 (0.69, 1.59)	0.93 (0.70, 1.23)	0.98 (0.68, 1.41)	
Home ownership (ref = owner)	2300	1	Tenant	1.05 (0.77, 1.42)	1.05 (0.85, 1.29)	1.02 (0.77, 1.34)	.97
		2	Tenant	1.06 (0.78, 1.43)	1.05 (0.85, 1.29)	1.02 (0.77, 1.34)	.96
		3	Tenant	0.92 (0.67, 1.26)	0.98 (0.80, 1.22)	0.98 (0.74, 1.30)	.96
		4	Tenant	1.01 (0.75, 1.38)	1.03 (0.84, 1.27)	.01 (0.77, 1.33)	.99
		5	Tenant	0.88 (0.64, 1.21)	0.97 (0.78, 1.20)	0.98 (0.74, 1.30)	.88
Lack of amenities (ref = lacks 0)	2326	1	Lacks 1 or more	1.22 (0.93, 1.62)	1.03 (0.85, 1.25)	0.99 (0.77, 1.27)	.53
		2	Lacks 1 or more	1.21 (0.92, 1.60)	1.03 (0.85, 1.24)	0.99 (0.77, 1.27)	.57
		3	Lacks 1 or more	1.12 (0.84, 1.48)	0.99 (0.82, 1.21)	0.97 (0.75, 1.25)	.85
		4	Lacks 1 or more	1.24 (0.94, 1.65)	1.04 (0.86, 1.26)	0.99 (0.77, 1.27)	.48
		5	Lacks 1 or more	1.14 (0.85, 1.52)	1.01 (0.83, 1.22)	0.98 (0.76, 1.26)	.81
Overcrowding (ref = not overcrowded)	2298	1	Overcrowded	1.20 (0.64, 2.27)	1.20 (0.77, 1.87)	0.72 (0.36, 1.44)	.47
		2	Overcrowded	1.12 (0.59, 2.11)	1.17 (0.75, 1.82)	0.72 (0.36, 1.44)	.56
		3	Overcrowded	1.06 (0.56, 2.03)	1.15 (0.73, 1.80)	0.70 (0.35, 1.42)	.58
		4	Overcrowded	1.06 (0.56, 2.03)	1.14 (0.73, 1.79)	0.71 (0.35, 1.43)	.60
		5	Overcrowded	1.01 (0.52, 1.95)	1.13 (0.72, 1.77)	0.71 (0.35, 1.44)	.64
Maternal education (ref = dip / degree)	2117	1	Secondary	1.02 (0.56, 1.87)	0.74 (0.50, 1.11)	0.57 (0.34, 0.96)	.42
			Primary	1.07 (0.64, 1.79)	0.81 (0.58, 1.13)	0.75 (0.50, 1.13)	
		2	Secondary	1.06 (0.58, 1.95)	0.76 (0.51, 1.13)	0.56 (0.34, 0.95)	.40
			Primary	1.08 (0.65, 1.82)	0.82 (0.59, 1.13)	0.75 (0.49, 1.13)	
		3	Secondary	1.04 (0.56, 1.91)	0.74 (0.50, 1.10)	0.57 (0.34, 0.96)	.34
			Primary	0.94 (0.55, 1.58)	0.75 (0.53, 1.04)	0.72 (0.48, 1.10)	

For peer review only

		4	Secondary	1.07 (0.58, 1.97)	0.76 (0.51, 1.13)	0.57 (0.34, 0.95)	.40
			Primary	1.11 (0.66, 1.88)	0.82 (0.59, 1.15)	0.75 (0.50, 1.13)	
		5	Secondary	1.01 (0.54, 1.88)	0.74 (0.50, 1.11)	0.57 (0.34, 0.97)	.38
			Primary	0.94 (0.55, 1.61)	0.76 (0.54, 1.06)	0.73 (0.48, 1.11)	
Paternal Education (ref = dip / degree)	2099	1	Secondary	1.08 (0.64, 1.81)	1.24 (0.87, 1.76)	0.83 (0.53, 1.30)	.71
			Primary	1.10 (0.73, 1.66)	1.07 (0.81, 1.42)	0.84 (0.60, 1.20)	
		2	Secondary	1.12 (0.66, 1.87)	1.26 (0.89, 1.78)	0.83 (0.53, 1.30)	.68
			Primary	1.11 (0.73, 1.67)	1.07 (0.81, 1.42)	0.84 (0.60, 1.20)	
		3	Secondary	1.03 (0.61, 1.75)	1.21 (0.85, 1.72)	0.81 (0.51, 1.27)	.60
			Primary	0.89 (0.58, 1.37)	0.97 (0.73, 1.30)	0.80 (0.56, 1.15)	
		4	Secondary	1.09 (0.64, 1.84)	1.24 (0.87, 1.77)	0.82 (0.52, 1.30)	.70
			Primary	1.09 (0.72, 1.66)	1.06 (0.80, 1.41)	0.84 (0.59, 1.19)	
		5	Secondary	0.99 (0.58, 1.69)	1.19 (0.84, 1.70)	0.80 (0.51, 1.27)	.64
			Primary	0.90 (0.58, 1.39)	0.97 (0.72, 1.30)	0.81 (0.57, 1.16)	

1. Unadjusted; 2. Adjusted for sex; 3. Adjusted for sex, BMI, smoking, alcohol, exercise; 4. Adjusted for sex and GHQ-28 caseness; 5. Adjusted for sex, BMI, smoking, alcohol, leisure-time physical activity, GHQ-28 caseness and marital status

For peer review only

Supplementary Table 5. Associations between indicators of socioeconomic position in earlier adulthood and pain at age 68.

Exposure	n	Model	Exposure level	CWP vs no pain RRR (95% CI)	CRP vs no pain RRR (95% CI)	Other pain vs no pain RRR (95% CI)	p
Education* Females (ref = degree)	1194	1	Secondary or less	1.10 (0.53, 2.27)	2.04 (1.08, 3.84)	0.54 (0.30, 0.98)	<.01
		3	Secondary or less	0.85 (0.40, 1.79)	1.83 (0.96, 3.46)	0.52 (0.28, 0.96)	<.01
		4	Secondary or less	1.22 (0.58, 2.57)	2.15 (1.14, 4.08);	0.56 (0.31, 1.03)	<.01
		5	Secondary or less	0.94 (0.44, 2.01)	1.93 (1.01, 3.69)	0.54 (0.29, 1.00)	<.01
Education* Males (ref = degree)	1103	1	Secondary or less	3.32 (1.41, 7.80)	1.11 (0.77, 1.59)	1.51 (0.93, 2.46)	<.01
		3	Secondary or less	2.64 (1.11, 6.32)	1.06 (0.73, 1.53)	1.45 (0.88, 2.39)	.06
		4	Secondary or less	3.35 (1.41, 7.93)	1.11 (0.78, 1.60)	1.51 (0.93, 2.47)	<.01
		5	Secondary or less	2.68 (1.12, 6.45)	1.07 (0.74, 1.56)	1.45 (0.88, 2.40)	.06
Financial hardship (ref = none)	2150	1	Minimal	1.40 (0.80, 2.46)	1.14 (0.76, 1.71)	0.98 (0.56, 1.71)	<.01
			Moderate	2.73 (1.45, 5.15)	1.34 (0.78, 2.33)	1.31 (0.64, 2.67)	
			Most	5.46 (2.62, 11.39)	1.83 (0.89, 3.74)	1.67 (0.67, 4.18)	
		2	Minimal	1.37 (0.78, 2.42)	1.13 (0.75, 1.70)	0.98 (0.56, 1.71)	<.01
			Moderate	2.61 (1.38, 4.95)	1.31 (0.76, 2.28)	1.31 (0.64, 2.67)	
			Most	5.38 (2.56, 11.30)	1.81 (0.89, 3.72)	1.67 (0.67, 4.18)	
		3	Minimal	1.29 (0.72, 2.28)	1.09 (0.72, 1.64)	0.95 (0.54, 1.66)	<.01
			Moderate	2.49 (1.30, 4.76)	1.33 (0.76, 2.31)	1.32 (0.65, 2.70)	
			Most	4.85 (2.28, 10.35)	1.74 (0.84, 3.58)	1.68 (0.67, 4.23)	
		4	Minimal	1.30 (0.73, 2.30)	1.10 (0.73, 1.66)	0.97 (0.55, 1.69)	<.01
			Moderate	2.44 (1.27, 4.67)	1.27 (0.73, 2.21)	1.29 (0.63, 2.63)	
			Most	4.96 (2.33, 10.57)	1.75 (0.85, 3.60)	1.65 (0.66, 4.13)	
		5	Minimal	1.21 (0.67, 2.17)	1.09 (0.72, 1.65)	0.95 (0.54, 1.68)	.03
			Moderate	2.32 (1.19, 4.52)	1.32 (0.75, 2.30)	1.34 (0.65, 2.74)	
			Most	4.44 (2.02, 9.77)	1.79 (0.86, 3.74)	1.75 (0.69, 4.47)	
Occupational class (ref = I-IIINM)	2219	1	IIINM	1.46 (1.03, 2.09)	1.06 (0.83, 1.36)	1.13 (0.83, 1.54)	0.09
			IIIM	1.25 (0.81, 1.92)	1.28 (0.97, 1.69)	0.98 (0.67, 1.43)	
			IV – V	1.80 (1.17, 2.75)	1.34 (0.99, 1.82)	0.92 (0.60, 1.42)	
		2	IIINM	1.15 (0.79, 1.66)	0.95 (0.73, 1.23)	1.16 (0.83, 1.61)	.15
			IIIM	1.41 (0.92, 2.19)	1.35 (1.02, 1.79)	0.97 (0.66, 1.41)	
			IV – V	1.52 (0.99, 2.34)	1.24 (0.91, 1.69)	0.94 (0.61, 1.45)	
		3	IIINM	1.09 (0.75, 1.59)	0.93 (0.71, 1.20)	1.14 (0.82, 1.59)	.44
			IIIM	1.22 (0.78, 1.90)	1.29 (0.96, 1.71)	0.94 (0.64, 1.39)	
			IV – V	1.26 (0.80, 1.97)	1.19 (0.87, 1.64)	0.93 (0.60, 1.44)	
		4	IIINM	1.11 (0.76, 1.62)	0.93 (0.71, 1.21)	1.15 (0.83, 1.60)	.17
			IIIM	1.45 (0.93, 2.25)	1.36 (1.02, 1.81)	0.97 (0.66, 1.42)	
			IV – V	1.44 (0.93, 2.23)	1.20 (0.88, 1.65)	0.93 (0.60, 1.43)	

For peer review only

		5	IIINM	1.08 (0.74, 1.59)	0.91 (0.70, 1.19)	1.13 (0.81, 1.58)	.38
			IIIM	1.29 (0.82, 2.03)	1.31 (0.98, 1.75)	0.93 (0.63, 1.38)	
			IV – V	1.22 (0.77, 1.92)	1.17 (0.85, 1.61)	0.93 (0.59, 1.44)	
Home ownership (ref = owner)	2294	1	Tenant	1.81 (1.21, 2.70)	1.07 (0.77, 1.48)	1.33 (0.89, 1.98)	.03
		2	Tenant	1.81 (1.21, 2.72)	1.07 (0.77, 1.48)	1.33 (0.89, 1.98)	.03
		3	Tenant	1.60 (1.06, 2.42)	1.02 (0.73, 1.41)	1.33 (0.89, 1.99)	.10
		4	Tenant	1.79 (1.19, 2.69)	1.06 (0.77, 1.47)	1.33 (0.89, 1.98)	.04
		5	Tenant	1.62 (1.06, 2.49)	1.06 (0.76, 1.48)	1.36 (0.90, 2.05)	.11

1. Unadjusted; 2. Adjusted for sex; 3. Adjusted for sex, BMI, smoking, alcohol and leisure-time physical activity; 4. Adjusted for sex and GHQ-28 caseness; 5. Adjusted for sex, BMI, smoking, alcohol, leisure-time physical activity, GHQ-28 caseness and marital status * The models for education are stratified by sex due to a significant interaction term ($p < 0.001$). Model 2 is therefore omitted and no adjustment for sex is made.

For peer review only

Supplementary Table 6. Associations between indicators of socioeconomic position in later adulthood and pain at age 68.

Exposure	n	Model	Exposure level	CWP vs no pain RRR (95% CI)	CRP v no pain RRR (95% CI)	Other pain vs no pain RRR (95% CI)	p
Home ownership (ref = owner)	2038	1	Tenant	1.17 (0.85, 1.62)	1.01 (0.81, 1.26)	0.90 (0.67, 1.22)	.62
		2	Tenant	1.25 (0.90, 1.73)	1.04 (0.83, 1.30)	0.90 (0.67, 1.22)	.44
		3	Tenant	1.08 (0.78, 1.52)	0.97 (0.77, 1.22)	0.88 (0.65, 1.19)	.74
		4	Tenant	1.20 (0.86, 1.67)	1.02 (0.81, 1.28)	0.90 (0.67, 1.21)	.55
		5	Tenant	1.07 (0.76, 1.51)	0.98 (0.78, 1.24)	0.89 (0.66, 1.21)	.83
Financial hardship (ref = none)	1892	1	Minimal	1.55 (0.60, 3.98)	0.87 (0.40, 1.92)	0.77 (0.26, 2.31)	.58
			Moderate	2.06 (0.88, 4.85)	1.74 (0.90, 3.35)	1.54 (0.66, 3.61)	
			Most	1.99 (0.68, 5.80)	1.48 (0.64, 3.43)	0.89 (0.25, 3.22)	
		2	Minimal	1.61 (0.62, 4.16)	0.88 (0.40, 1.94)	0.77 (0.26, 2.30)	.52
			Moderate	2.03 (0.86, 4.80)	1.73 (0.90, 3.34)	1.54 (0.66, 3.62)	
			Most	2.26 (0.77, 6.64)	1.53 (0.66, 3.57)	0.89 (0.24, 3.20)	
		3	Minimal	1.49 (0.57, 3.94)	0.84 (0.38, 1.86)	0.76 (0.25, 2.28)	.64
			Moderate	1.79 (0.74, 4.28)	1.64 (0.85, 3.18)	1.52 (0.64, 3.58)	
			Most	2.35 (0.79, 7.01)	1.51 (0.64, 3.53)	0.89 (0.24, 3.24)	
		4	Minimal	1.48 (0.56, 3.89)	0.84 (0.38, 1.86)	0.75 (0.25, 2.26)	.86
			Moderate	1.61 (0.67, 3.88)	1.54 (0.79, 2.99)	1.46 (0.62, 3.44)	
			Most	1.67 (0.55, 5.04)	1.31 (0.56, 3.09)	0.82 (0.23, 3.00)	
		5	Minimal	1.39 (0.51, 3.74)	0.83 (0.37, 1.87)	0.77 (0.25, 2.34)	.89
			Moderate	1.47 (0.60, 3.63)	1.56 (0.79, 3.05)	1.49 (0.63, 3.54)	
			Most	1.71 (0.55, 5.29)	1.39 (0.58, 3.32)	0.91 (0.25, 3.34)	

1. Unadjusted; 2. Adjusted for sex; 3. Adjusted for sex, BMI, smoking, alcohol and leisure-time physical activity; 4. Adjusted for sex and GHQ-28 caseness; 5. Adjusted for sex, BMI, smoking, alcohol, leisure-time physical activity, GHQ-28 caseness and marital status

For peer review only

Supplementary Table 7. Changes in SEP measures between early and later time periods.

	n (%)	
Tenant status (n = 1,956)	*Tenant status in later adulthood*	
Tenant status in childhood	Owner-occupier	Tenant
Owner-occupier	471 (23.8)	127 (6.4)
Tenant	931 (47.1)	446 (22.6)
Financial hardship (n = 1,795)	*Financial hardship later adulthood*	
Financial hardship earlier adulthood	No (none or minimal)	Yes (moderate or most)
No (none or minimal)	1637 (91.2)	52 (2.9)
Yes (moderate or most)	84 (4.7)	22 (1.2)

For peer review only

Supplementary Table 8. Accumulation of occupational class and financial hardship.

Accumulation	Tenant status	Financial hardship
	n (%)	
0 (high at both time points)	453 (23.2)	1,637 (91.2)
1	1017 (52.0)	136 (7.6)
2	352 (18.0)	22 (1.2)
3	134 (6.9)	-
Unknown	422	583

For peer review only

Supplementary Table 9. Associations between cumulative exposure to tenant status and financial hardship and pain at age 68.

Exposure	n	Model	Exposure level	CWP vs no pain RRR (95% CI)	CRP v no pain RRR (95% CI)	Other pain vs no pain RRR (95% CI)	p
Financial hardship accumulation (ref = No or minimal hardship)	1795	1	Hardship at one point	2.23 (1.32, 3.78)	1.36 (0.88, 2.08)	1.57 (0.93, 2.65)	<.01
			Hardship at both	5.44 (1.80, 16.39)	1.77 (0.59, 5.28)	1.14 (0.23, 5.68)	
		2	Hardship at one point	2.26 (1.33, 3.83)	1.36 (0.89, 2.08)	1.57 (0.93, 2.65)	<.01
			Hardship at both	5.18 (1.70, 15.77)	1.74 (0.58, 5.20)	1.13 (0.23, 5.66)	
		3	Hardship at one point	2.17 (1.27, 3.71)	1.35 (0.88, 2.08)	1.59 (0.93, 2.70)	.02
			Hardship at both	4.45 (1.42, 13.94)	1.63 (0.54, 4.92)	1.12 (0.22, 5.64)	
		4	Hardship at one point	1.99 (1.16, 3.41)	1.28 (0.83, 1.97)	1.53 (0.90, 2.59)	.05
			Hardship at both	4.26 (1.36, 13.32)	1.57 (0.52, 4.76)	1.09 (0.22, 5.45)	
		5	Hardship at one point	1.93 (1.11, 3.35)	1.34 (0.86, 2.08)	1.59 (0.93, 2.73)	.09
			Hardship at both	3.90 (1.20, 12.64)	1.60 (0.52, 4.92)	1.15 (0.23, 5.84)	
Housing ownership accumulation (ref = always owner)	1956	1	Tenant at one point	0.90 (0.61, 1.32)	0.91 (0.70, 1.18)	0.89 (0.63, 1.25)	.47
			Tenant at two points	1.02 (0.63, 1.65)	1.05 (0.76, 1.45)	0.95 (0.62, 1.47)	
			Tenant at all points	1.96 (1.09, 3.52)	1.13 (0.71, 1.80)	1.16 (0.64, 2.11)	
		2	Tenant at one point	0.90 (0.61, 1.33)	0.91 (0.70, 1.18)	0.89 (0.63, 1.25)	.43
			Tenant at two points	1.10 (0.68, 1.79)	1.08 (0.78, 1.50)	0.96 (0.62, 1.47)	
			Tenant at all points	1.99 (1.10, 3.59)	1.13 (0.71, 1.81)	1.17 (0.64, 2.11)	
		3	Tenant at one point	0.84 (0.56, 1.24)	0.86 (0.66, 1.12)	0.86 (0.61, 1.21)	.73
			Tenant at two points	0.93 (0.56, 1.53)	0.98 (0.70, 1.37)	0.90 (0.58, 1.39)	
			Tenant at all points	1.50 (0.81, 2.78)	0.97 (0.60, 1.57)	1.07 (0.58, 1.98)	
		4	Tenant at one point	0.89 (0.60, 1.32)	0.91 (0.70, 1.18)	0.89 (0.63, 1.25)	.60
			Tenant at two points	1.05 (0.64, 1.72)	1.06 (0.76, 1.47)	0.95 (0.62, 1.46)	
			Tenant at all points	1.83 (1.00, 3.34)	1.10 (0.69, 1.76)	1.15 (0.64, 2.09)	
		5	Tenant at one point	0.83 (0.55, 1.23)	0.85 (0.65, 1.11)	0.86 (0.61, 1.21)	.72
			Tenant at two points	0.90 (0.54, 1.49)	0.99 (0.71, 1.39)	0.92 (0.59, 1.43)	
			Tenant at all points	1.48 (0.78, 2.78)	0.99 (0.61, 1.60)	1.12 (0.60, 2.08)	

1. Unadjusted; 2. Adjusted for sex; 3. Adjusted for sex, BMI, smoking, alcohol and leisure-time physical activity; 4. Adjusted for sex and GHQ-28 caseness; 5. Adjusted for sex, BMI, smoking, alcohol, leisure-time physical activity, GHQ-28 caseness and marital status

For peer review only

STROBE 2007 (v4) checklist of items to be included in reports of observational studies in epidemiology*

Checklist for cohort, case-control, and cross-sectional studies (combined)

Section/Topic	Item #	Recommendation	Reported on page #
**Title and abstract**	1	(*a*) Indicate the study's design with a commonly used term in the title or the abstract	1
		(*b*) Provide in the abstract an informative and balanced summary of what was done and what was found	3
**Introduction**			
Background/rationale	2	Explain the scientific background and rationale for the investigation being reported	5-6
Objectives	3	State specific objectives, including any pre-specified hypotheses	6
**Methods**			
Study design	4	Present key elements of study design early in the paper	7
Setting	5	Describe the setting, locations, and relevant dates, including periods of recruitment, exposure, follow-up, and data collection	7-8
Participants	6	(*a*) *Cohort study*—Give the eligibility criteria, and the sources and methods of selection of participants. Describe methods of follow-up<br>*Case-control study*—Give the eligibility criteria, and the sources and methods of case ascertainment and control selection. Give the rationale for the choice of cases and controls<br>*Cross-sectional study*—Give the eligibility criteria, and the sources and methods of selection of participants	7
		(*b*) *Cohort study*—For matched studies, give matching criteria and number of exposed and unexposed<br>*Case-control study*—For matched studies, give matching criteria and the number of controls per case	N/A
Variables	7	Clearly define all outcomes, exposures, predictors, potential confounders, and effect modifiers. Give diagnostic criteria, if applicable	8-9 & Table 1
Data sources/ measurement	8*	For each variable of interest, give sources of data and details of methods of assessment (measurement). Describe comparability of assessment methods if there is more than one group	8-9
Bias	9	Describe any efforts to address potential sources of bias	10-11
Study size	10	Explain how the study size was arrived at	7
Quantitative variables	11	Explain how quantitative variables were handled in the analyses. If applicable, describe which groupings were chosen and why	9-10
Statistical methods	12	(*a*) Describe all statistical methods, including those used to control for confounding	10
		(*b*) Describe any methods used to examine subgroups and interactions	10
		(*c*) Explain how missing data were addressed	10-11
		(*d*) *Cohort study*—If applicable, explain how loss to follow-up was addressed<br>*Case-control study*—If applicable, explain how matching of cases and controls was addressed	N/A

For peer review only

		Cross-sectional study—If applicable, describe analytical methods taking account of sampling strategy	
		(*e*) Describe any sensitivity analyses	11
**Results**			
Participants	13*	(a) Report numbers of individuals at each stage of study—eg numbers potentially eligible, examined for eligibility, confirmed eligible, included in the study, completing follow-up, and analysed	12
		(b) Give reasons for non-participation at each stage	7
		(c) Consider use of a flow diagram	N/A
Descriptive data	14*	(a) Give characteristics of study participants (eg demographic, clinical, social) and information on exposures and potential confounders	12
		(b) Indicate number of participants with missing data for each variable of interest	10 & Table 2
		(c) *Cohort study*—Summarise follow-up time (eg, average and total amount)	7-9
Outcome data	15*	*Cohort study*—Report numbers of outcome events or summary measures over time	12 & Table 2
		Case-control study—Report numbers in each exposure category, or summary measures of exposure	N/A
		Cross-sectional study—Report numbers of outcome events or summary measures	N/A
Main results	16	(*a*) Give unadjusted estimates and, if applicable, confounder-adjusted estimates and their precision (eg, 95% confidence interval). Make clear which confounders were adjusted for and why they were included	12-13 & Tables 3 to 5
		(*b*) Report category boundaries when continuous variables were categorized	9 (GHQ-28 scores)
		(*c*) If relevant, consider translating estimates of relative risk into absolute risk for a meaningful time period	N/A
Other analyses	17	Report other analyses done—eg analyses of subgroups and interactions, and sensitivity analyses	12-13 & Tables 3 to 5
**Discussion**			
Key results	18	Summarise key results with reference to study objectives	14
Limitations	19	Discuss limitations of the study, taking into account sources of potential bias or imprecision. Discuss both direction and magnitude of any potential bias	16
Interpretation	20	Give a cautious overall interpretation of results considering objectives, limitations, multiplicity of analyses, results from similar studies, and other relevant evidence	16-17
Generalisability	21	Discuss the generalisability (external validity) of the study results	14-15
**Other information**			
Funding	22	Give the source of funding and the role of the funders for the present study and, if applicable, for the original study on which the present article is based	18

*Give information separately for cases and controls in case-control studies and, if applicable, for exposed and unexposed groups in cohort and cross-sectional studies.

Note: An Explanation and Elaboration article discusses each checklist item and gives methodological background and published examples of transparent reporting. The STROBE checklist is best used in conjunction with this article (freely available on the Web sites of PLoS Medicine at http://www.plosmedicine.org/, Annals of Internal Medicine at http://www.annals.org/, and Epidemiology at http://www.epidem.com/). Information on the STROBE Initiative is available at www.strobe-statement.org.

For peer review only

BMJ Open

Lifetime socioeconomic circumstances and chronic pain in later adulthood: findings from a British birth cohort study

Journal:	*BMJ Open*
Manuscript ID	bmjopen-2018-024250.R1
Article Type:	Research
Date Submitted by the Author:	02-Oct-2018
Complete List of Authors:	Jay, Matthew; University College London, GOS Institute of Child Health; Great Ormond Street Hospital For Children NHS Foundation Trust, Department of Anaesthesia and Pain Medicine<br>Bendayan, Rebecca; King's College London, Institute of Psychiatry, Psychology and Neuroscience, Dept. Biostatistics and Health Informatics; University College London, MRC Unit for Lifelong Health and Ageing at UCL<br>Cooper, Rachel; University College London, MRC Unit for Lifelong Health and Ageing<br>Muthuri, Stella; MRC Unit for Lifelong Health and Ageing at UCL
**Primary Subject Heading**:	Epidemiology
Secondary Subject Heading:	Geriatric medicine, Public health
Keywords:	EPIDEMIOLOGY, PAIN MANAGEMENT, PUBLIC HEALTH

SCHOLARONE™
Manuscripts

Lifetime socioeconomic circumstances and chronic pain in later adulthood: findings from a British birth cohort study

Matthew A Jay[1,2*]

Rebecca Bendayan[3,4]

Rachel Cooper[3]

Stella Muthuri[3]

1: UCL Great Ormond Street Institute of Child Health: Population, Policy & Practice Programme

2: Great Ormond Street Hospital for Children NHS Foundation Trust: Department of Anaesthesia and Pain Medicine

3: MRC Unit for Lifelong Health and Ageing at UCL

4: Dept. Biostatistics and Health Informatics

Institute of Psychiatry, Psychology & Neuroscience (IoPPN)

King's College London

*Corresponding author: matthew.jay.15@ucl.ac.uk

UCL GOS Institute of Child Health

30 Guilford Street

London WC1N 1DT

+44 (0)20 3549 5030

For peer review only - http://bmjopen.bmj.com/site/about/guidelines.xhtml

CONTRIBUTOR STATEMENT

Matthew A Jay (MAJ) had full access to the data and takes full responsibility for the integrity and accuracy of analysis. MAJ, Stella G Muthuri (SGM) and Rebecca Bendayan (RB) conceived the idea for this study. MAJ, SGM, RB and Rachel Cooper contributed to study design. MAJ analysed the data and drafted the manuscript. All authors contributed critically to the manuscript and provided final approval of the version to be published.

For peer review only

For peer review only - http://bmjopen.bmj.com/site/about/guidelines.xhtml

ABSTRACT

Objectives: To investigate associations between a range of different indicators of socioeconomic position (SEP: occupational class, education, household overcrowding and tenure, and experience of financial hardship) across life and chronic widespread and regional pain (CWP and CRP) at age 68.

Design: Prospective birth cohort; the MRC National Survey of Health and Development.

Setting: England, Scotland and Wales.

Participants: Up to 2378 men and women who have been followed up since birth in 1946 to age 68.

Primary outcome measures: Based on their self-report of pain at age 68, participants were classified as: CWP (American College of Rheumatology criteria), CRP (pain of at least 3 months' duration but which does not meet definition of CWP), other pain (<3 months in duration) or no pain.

Results: At age 68, the prevalence of CWP was 13.3% in women and 7.8% in men and that of CRP was 32.3% and 28.7% in women and men, respectively. There was no clear evidence that indicators of SEP in childhood or later adulthood were associated with pain. Having experienced (*vs* not) financial hardship and being a tenant (*vs* owner-occupier) in earlier adulthood were both associated with an increased risk of CWP; for example, moderate hardship adjusted relative risk ratio (RRR_{adj}) 2.32 (95% CI 1.19, 4.52); most hardship RRR_{adj} 4.44 (95% CI 2.02, 9.77). Accumulation of financial hardship across earlier and later adulthood was also associated with an increased risk of CWP.

Conclusions: Consideration of socioeconomic factors in earlier adulthood may be important when identifying targets for intervention to prevent CWP in later life.

For peer review only - http://bmjopen.bmj.com/site/about/guidelines.xhtml

ARTICLE SUMMARY

Strengths and limitations of this study

- We used data from a sample of older adults in Great Britain who were representative of the general population born at a similar time.
- Studying a wide range of indicators of socioeconomic position (SEP) over a 60 year period provided a unique insight into the associations between SEP and chronic widespread and regional pain at age 68.
- Prospective data collection across life reduced the risk of misclassification bias.
- The study might have been subject to survivor bias with the result that associations may be biased towards the null.

For peer review only

For peer review only - http://bmjopen.bmj.com/site/about/guidelines.xhtml

INTRODUCTION

Chronic widespread and regional pain (CWP and CRP) are leading causes of disability globally[1] particularly in later adulthood.[2] CWP is defined according to the American College of Rheumatology (ACR) criteria[3] as pain present for 3 months or longer, both above and below the waist; on both the left and right side of the body; and in the axial skeleton. CRP is any pain present for 3 months or longer which does not otherwise meet these ACR criteria. In a systematic review, the prevalence of chronic pain among adults in the UK was estimated to be 44% (95% CI 38%, 49%) and that of CWP to be 14% (95% CI 12%, 16%),[4] an estimate which is similar in other countries.[5 6] Understanding risk factors for CWP and CRP, such as socioeconomic position (SEP) on which there is currently limited population-level evidence,[7] is therefore important to develop preventive public health strategies. Pain is a biopsychosocial phenomenon, meaning that it is known to be affected by a range biological, psychological and social factors.[8] SEP is therefore an important target for study and potential intervention.

Few studies have examined associations between SEP and CWP, CRP or a combined measure of chronic pain; they have largely been cross-sectional, tending to treat SEP as a confounder.[9-14] They mostly examine only one or two indicators of SEP despite its multifaceted nature[15-17] with different aspects of SEP potentially related to CWP and CRP in different ways. Few also consider the role of SEP across life.[18-20] Of those that do, Macfarlane et al (2009)[18] and Power et al (2007),[19] using the 1958 British Birth Cohort, found associations between lower SEP (paternal and own occupational class during childhood and earlier adulthood, respectively) and higher risk of CWP at age 44-45. Macfarlane et al (2009)[18] also found lower occupational class was associated with an increased risk of CRP at specific sites; many of these associations, however, were attenuated

For peer review only - http://bmjopen.bmj.com/site/about/guidelines.xhtml

after adjustment. Goosby (2013),[20] using the US Health and Retirement Study, found higher education and income were associated with lower risk of chronic pain (i.e. arthritis/rheumatism; back or neck problems or received treatment in the past 12 months; frequent or severe headaches; any other chronic pain) in people aged 18 to 64. The extent to which SEP across life is associated with CWP or CRP in participants beyond midlife remains to be determined. Taking a life course approach, by investigating SEP at different life stages, could help identify new opportunities for intervention as accumulation of exposure to social stressors across life may be associated with an increased risk of CWP and CRP at older ages.[21]

There are important gaps in knowledge. Firstly, the multifaceted nature of SEP has been neglected in research into the association between SEP, CWP and CRP. Our first aim was therefore to investigate whether a range of different SEP indicators across childhood and adulthood were associated with CWP, CRP or other pain at age 68. Examining CWP and CRP is important as they may have different underlying aetiologies. Secondly, associations between SEP and CWP or CRP in later adulthood cannot be fully understood without considering how SEP operates across life. We therefore also aimed to test whether observed associations are cumulative. We hypothesised that (1) lower levels of each SEP indicator would be associated with an increased risk of CWP (but not necessarily CRP on the basis of the weaker associations previously observed[18]) in later adulthood and (2) that accumulation of low SEP across life would also be associated with increased risk of CWP.

For peer review only - http://bmjopen.bmj.com/site/about/guidelines.xhtml

METHODS

Sample

Data were drawn from the Medical Research Council National Survey of Health and Development (NSHD), a nationally representative[22 23] longitudinal study of a sample (n=5,362) of single births during the first week of March 1946 in Great Britain.[24] Data collection occurred approximately every two years during childhood and every five to ten years during adulthood, with 24 data collection rounds up to 2014-2015.[22 24 25] Details on overall participation rates, which were generally high, are available elsewhere.[22 26] Of the 2,942 people in the target sample for the most recent data collection at age 68, in 2014-2015, 2,453 (83.4%) completed a postal questionnaire. No attempt was made to contact the remaining 2,420 study members: 957 (17.8%) had already died, 620 (11.6%) had previously withdrawn from the study, 448 (8.3%) had emigrated and were no longer in contact with the study, and 395 (7.4%) had been untraceable for more than five years. Participants who responded were eligible for the present study if they had provided responses to questions on pain (n = 2,378).

Ethical approval was received for each data collection; in 2014-2015 this was obtained from the Queen Square Ethics Committee (14/LO/1073) and the Scotland A Research Ethics Committee (14/SS/1009) and written informed consent was obtained from study members.

Patient and Public involvement statement

Participants have a lifelong association with NSHD. Over the 70 years of the study, the research team has increasingly involved participants, in line with changing norms about conducting cohort studies, starting at age 16 (in 1962) with the annual dissemination of study findings in birthday cards. Participants receive personal letters whenever they raise queries or

For peer review only - http://bmjopen.bmj.com/site/about/guidelines.xhtml

provide additional comments, including suggestions for new topics to study. In the last ten years, the research team has increased the level of participant involvement through invitations to study events (65th and 70th birthday celebrations), public engagement activities, and focus groups to discuss clinical sub-studies. When piloting new questionnaires and assessments, we recruit patients from GP practices or from the UCLH Patient Public Involvement (PPI) group, and take into account their feedback when designing the mainstage fieldwork.

Ascertainment of pain at age 68

Participants were asked: 'In the *last month*, have you had any ache or pain which has lasted for *one day or longer*? (Please do not include pain occurring only during the course of a feverish illness such as flu) [emphasis in original].' Those who answered yes were then asked whether they had been aware of this pain for at least 3 months and were asked to shade on a 4-view body manikin all the areas where they felt pain. Responses to these questions were used to classify participants as having CWP (defined according to the ACR criteria[3]). Those who reported pain of at least three months' duration but did not meet the CWP definition were classified as CRP and those reporting pain of less than three months duration were categorised as having other pain.[27]

Indicators of socioeconomic position

A range of SEP indicators were selected for study *a priori* in order to capture different facets of the SEP construct including psychosocial as well as material aspects.[16 17] These indicators were assessed prospectively across life and for the purposes of these analyses were grouped into three life stages (childhood [age 0 to 15]; earlier adulthood [age 26 to 53] and later adulthood [age 60 to 64]). Indicators of childhood SEP were father's occupational class, paternal and maternal education, household overcrowding, housing tenure, and lack of

For peer review only - http://bmjopen.bmj.com/site/about/guidelines.xhtml

household amenities. Where possible, measures were taken at age 4, except for lack of amenities which was ascertained at age 2. However, if data were missing at age 4, comparable measures at ages 11 or 15, were used. See Table 1 for details of categorisation of each measure.

Indicators of SEP in earlier adulthood (i.e. 26 to 53) were highest educational level attained by age 26, financial hardship at age 43 and own occupational class and home ownership at age 53 (or at 43 or 36 if missing at 53) (see Table 1). Indicators of SEP in later adulthood (age 60-64) were home ownership and financial hardship (see Table 1).

Covariates

Potential confounders were identified from the existing literature.[10 18 28-31] BMI (kg·m^{-2}) was calculated using measures of height and weight recorded by trained nurses at age 60-64. Cigarette smoking status (current, ex or never), participation in sports or vigorous leisure activities in the previous 4 weeks (inactive, 1-4 times per month or 5 or more times per month) and marital status (never married, married/civil partnership, separated/divorced, widowed) were all self-reported at age 68. Participants were asked how often they consumed alcoholic drinks in the last year and to record the number of alcoholic drinks (beers, wines and spirits) they drank in the last 7 days. Units were summed to create an overall measure of alcohol consumptions in units in the previous week at age 68. Affective symptoms were measured using General Health Questionnaire-28 (GHQ-28)[32] score at age 60-64. Items were scored 0-0-1-1, then summed and caseness was identified based on a cut-off of 5 or more.[32]

For peer review only - http://bmjopen.bmj.com/site/about/guidelines.xhtml

Statistical methods

To examine the associations of each SEP indicator with CWP, CRP and other pain at age 68, a series of multinomial logistic regression models were specified. For each SEP indicator, a crude model was first constructed and adjusted for sex. An interaction term between each SEP indicator and sex was entered and subsequent models were sex-stratified if this term was statistically significant (likelihood ratio test, $p < .05$). Categories of covariates were entered as follows. First, BMI and health behaviours (smoking, physical activity and alcohol consumption) were entered. Secondly, adjustment was made for affective symptoms (GHQ-28 caseness). Finally a fully adjusted model which included all covariates was estimated.

To ascertain whether accumulation of low levels of SEP was associated with CWP, CRP or other pain, we derived ordinal indices that indicated, at each time point, whether the participant was in lower or higher SEP (these scores assume equal weight should be given to SEP at each life stage). We only generated these indices for indicators of SEP that had been found at any life stage to be associated with CWP, CRP or other pain and which had been measured at multiple life stages. These indices were entered into regression models and adjustments were carried out in the same order as above.

Models were fit with maximum likelihood and results are presented as relative risk ratios (RRR) with 95% confidence intervals (CIs). Statistical significance of each SEP indicator overall in each model was assessed using the likelihood ratio test. Where covariates were missing (GHQ-28 score [n = 494, 20.8%]; BMI [n = 472, 19.8%]; alcohol consumption [n = 243, 10.2%]; marital status [n = 45, 1.9%]; and smoking status [n = 22, 0.9%]), these were imputed using multiple imputation with chained equations[33] with 40 imputed datasets. The outcome and exposure variables were included in the imputation model but not themselves

For peer review only - http://bmjopen.bmj.com/site/about/guidelines.xhtml

imputed.[34] Parameter estimates were combined using Rubin's rules. Sensitivity analyses exploring these models with only complete cases were performed.

All data management and analysis was conducted in R[35] with RStudio[36] and the packages *mice*[33] and *nnet*.[37]

For peer review only

For peer review only - http://bmjopen.bmj.com/site/about/guidelines.xhtml

RESULTS

The characteristics of the 2,378 participants included in analyses are presented in Table 2. The prevalence of CWP among women and men was 13.3% and 7.8%, respectively. In women, the prevalence of CRP was 32.3% and other pain 12.5%. In men, these figures were 28.7% and 15.2%, respectively. The number and proportion of individuals in the lowest SEP categories is given in Table 2, with a full break-down by sex in Supplementary Tables 1 to 3.

There was no evidence of associations between any of the indicators of childhood SEP and CWP, CRP or other pain at age 68 (Table 3; models at all stages of adjustment in Supplementary Table 4).

Table 4 (and Supplementary Table 5) shows the RRRs of CWP, CRP and other pain (vs no pain) obtained from regression models for each indicator of SEP in earlier adulthood. There were sex differences in the associations between educational level and pain (education-sex interaction, $p<.01$). Women with lower levels of education were more likely to report CRP at age 68 compared to those with a degree or higher (RRR 1.93 [95% CI 1.01, 3.69]) and were less likely to report other pain (RRR 0.54, [0.29, 1.00]). Men with lower educational levels were more likely to report CWP than men with a degree or higher (2.68 [95% CI 1.12, 6.45]). Financial hardship in earlier adulthood was associated with higher risk of CWP at age 68; fully-adjusted RRR of CWP was 1.21 (95% CI 0.67, 2.17) for minimal hardship, 2.32 (95% CI 1.19, 4.52) for moderate hardship and 4.44 (95% CI 2.02, 9.77) for most financial hardship, compared with no financial hardship. Similar trends were observed for CRP and other pain but the RRRs were smaller and not statistically significant. In a sex-adjusted model, there was some evidence of association between the lowest occupational classes and an increased risk of CWP (RRR 1.52 [95% CI 0.99, 2.34]) which was attenuated in the fully-

For peer review only - http://bmjopen.bmj.com/site/about/guidelines.xhtml

adjusted model (RRR 1.22 [95% CI 0.77, 1.92]). Finally, being a tenant (*vs* being an owner-occupier) was associated with an increased risk of CWP at age 68. In a sex-adjusted model the RRR was 1.81 (95% CI 1.21, 2.72), which was slightly attenuated to 1.62 (1.06, 2.49) after adjustment for all covariates.

Neither indicator of SEP in later adulthood (home ownership and financial hardship) was associated with pain at age 68 (Table 4 and Supplementary Table 6).

As tenant status and financial hardship were the only two variables measured at multiple time points that were associated with CWP or CRP, these two were taken forward into the accumulation analyses. The number and proportion of participants in each group at the earliest and latest time points are cross-tabulated in Supplementary Table 7. Supplementary Table 8 shows the number and proportion of participants at each level of the derived accumulation indices.

Results from multinomial logistic regression models examining the cumulative associations between tenant status, financial hardship and CWP, CRP and other pain are shown in Table 5 (and Supplementary Table 9). These show that accumulation of tenant status over life was not associated with pain at age 68. By contrast, accumulation of financial hardship across adulthood was associated with an increased risk of CWP (hardship at one point reported *vs* no hardship RRR 1.93 [95% CI 1.11, 3.35]; hardship at both points RRR 3.90 [95% CI 1.20, 12.64]).

For peer review only - http://bmjopen.bmj.com/site/about/guidelines.xhtml
For peer review only

Sensitivity analyses revealed no major differences to the models fit using imputed data (directions of associations were the same and magnitudes were similar) and are not therefore presented.

For peer review only

For peer review only - http://bmjopen.bmj.com/site/about/guidelines.xhtml

DISCUSSION

We aimed to investigate whether different SEP indicators at different life stages were associated with risk of reporting CWP, CRP or other pain at age 68. Experience of financial hardship and tenant status in earlier adulthood were both associated with an increased risk of CWP at age 68. Lower educational attainment was also associated with an increased risk of CWP in men and an increased risk of CRP in women. There was no evidence of associations between any SEP indicator in childhood or later adulthood and any pain outcome at age 68. Our second aim was to assess whether observed associations were cumulative across different life stages and we found that there was a cumulative association between financial hardship during earlier and later adulthood and increased risk of CWP at age 68.

Our finding of stronger and more consistent associations between indicators of SEP in earlier adulthood and pain than with indicators of SEP in childhood or later adulthood, suggests that earlier adulthood may be a sensitive period. Financial hardship appears to be particularly important. This variable captures both subjective and objective elements of hardship (i.e., feeling unable to manage or being unable to pay bills, or both). There may therefore be a combination of psychosocial and material elements that are associated with an increased risk of CWP. It is also especially interesting that financial hardship in later adulthood was not independently associated with CWP but when combined with the same variable in earlier adulthood, a clear inverse association was observed. This suggests that, despite the lack of strong evidence of an association between financial hardship in later adulthood and CWP at 68, intervening to reduce the burden of hardship in later as well as earlier adulthood may prove beneficial in reducing the prevalence of CWP in older age.

For peer review only - http://bmjopen.bmj.com/site/about/guidelines.xhtml

The interaction between education and sex also requires explanation. An increased risk of CWP or CRP with lower levels of education is in line with our initial hypotheses but it is not clear why lower education was associated with an increased risk of CWP among men but with increased risk of CRP among women. The results may be partly explained by the fact that the binary categorisation of education used in our analyses captured more of the relevant variability in educational attainment among men than women: in our sample, 6.5% of women had a degree or higher compared with 17.2% of men (Supplementary Table 2). In addition, it may be the case that among this generation, comparing those who have a degree or higher with those with a lower level of educational attainment provides a more appropriate level of differentiation between higher and lower SEP among men than women. Previous work using the NSHD has shown that the normative work-family life course patterns for women was one of forming families earlier in life when compared with men.[38 39] Therefore, among this generation, achieving a university degree may have been less likely to provide women than men with access to resources (such as better remunerated work) associated with a lower risk of CWP.

Although we found evidence that earlier adulthood may be a sensitive period, it is also clear that choice of indicator is important as there was limited evidence of an association between occupational class and CWP or CRP. This is surprising given previous findings from the 1958 British Birth Cohort.[18 19] These previous analyses showed that lower parental occupational class in childhood and own occupational class at age 42 were associated with an increased risk of CWP at age 45 and that the association between own occupational class at 42 and CWP persisted after adjustment for parental occupational class in childhood (and in the case of Macfarlane et al (2009),[18] adjustment for potential confounders/mediators). Reasons for the discrepancy with our findings may be attributable to differences in study

For peer review only - http://bmjopen.bmj.com/site/about/guidelines.xhtml

design, particularly the ages at which pain was measured. Additionally, due to social, political and cultural changes over the course of the twentieth century, conditions at the time of birth in each of the cohorts were different; for example participants in the NSHD were born in 1946, which was the period immediately after World War II when food rationing was still in effect and important changes in housing regulations had not yet been passed, and so will have experienced poorer absolute economic and environmental conditions in early life than the cohort born in 1958. Further, the effects of occupation may act only contemporaneously in the aetiology of CWP, for example through the immediate psychosocial or physical demands of the work.[16 17 40 41]

Goosby[20] found that childhood conditions (i.e. self-reported parental education, receipt of financial assistance and having gone hungry) were associated with chronic pain prevalence in adulthood (ages 25-64). Differences between this study's findings and our own could be attributable to differences in the indicators of childhood socioeconomic circumstances used. In addition, the associations for childhood SEP indicators observed by Goosby[20] were somewhat attenuated in models that included adulthood SEP (income, education and benefit receipt since age 18). The latter were all associated with chronic pain in the expected directions. The fact that receipt of benefits was associated with chronic pain in Goosby's study[20] is consistent with our finding that financial hardship was associated with CWP.

This study has a number of strengths. The NSHD is largely representative of the population of older adults in Great Britain despite some losses to follow up,[24 26] meaning that results are likely to be generalisable to this generation. We examined a wide range of SEP indicators with data being collected over a 60 year period. The study is subject to very limited (if any) recall bias as data were collected prospectively. There is also therefore limited risk of

For peer review only - http://bmjopen.bmj.com/site/about/guidelines.xhtml

exposure or outcome misclassification (i.e. participants being incorrectly labelled as lower or higher SEP).

Some limitations are also noted. It was not possible to examine incident pain as no data on CWP or CRP had been ascertained before age 68. As onset of CWP and CRP may occur earlier in life and persist into later adulthood, future work will need to establish the temporality of associations. Although we adjusted for a range of covariates, we only did so at one time point. Changes in these variables may be associated with changes in pain state over time and failing to account for their time-varying nature might have resulted in residual confounding. There might also be survivor bias as lower SEP in childhood was associated with lower survival rates into adulthood in NSHD.[42] If so, this would bias associations towards the null and could partially explain why there was no association between childhood SEP and CWP or CRP at age 68. Finally, results might not be generalizable to younger, more ethnically diverse cohorts so the study should be replicated with subsequent generations, particularly the 1958 British Birth Cohort as participants in that study age, which also contains data on SEP and pain.

These results have important implications for public policy. Given its high prevalence, cost and associated disability, prevention of CWP and CRP should be considered a key public health priority. Policy makers should ensure that socioeconomic adversity across life is addressed in public health interventions, particularly the role that housing, education and financial hardship may play.

For peer review only - http://bmjopen.bmj.com/site/about/guidelines.xhtml

ACKNOWLEDGMENTS

The authors are grateful to NSHD study members for their continuing participation in the study. We also thank members of the NSHD scientific and data collection teams.

FUNDING STATEMENT

This work was supported by the UK Medical Research Council (MRC) which provides core funding for the MRC National Survey of Health and Development and supported SGM and RC (programme code: MC_UU_12019/4). SGM was also supported by MRC grant MR/L010399/1. MAJ was supported while carrying out this work by a grant from Great Ormond Street Hospital for Children NHS Foundation Trust. RB was supported in part by grant MR/R016372/1 for the King's College London MRC Skills Development Fellowship programme funded by the UK Medical Research Council and by grant IS-BRC-1215-20018 for the National Institute for Health Research (NIHR) Biomedical Research Centre at South London and Maudsley NHS Foundation Trust and King's College London. The views expressed are those of the authors and not necessarily those of the MRC, NHS, NIHR or the Department of Health and Social Care. The funders of the study had no role in the study design, data collection, data analysis, data interpretation, writing of the report or the decision to submit the article for publication.

COMPETING INTERESTS

The authors have no conflicts of interest to declare.

For peer review only - http://bmjopen.bmj.com/site/about/guidelines.xhtml

DATA SHARING STATEMENT

Data used in this publication are available to bona fide researchers upon request to the NSHD Data Sharing Committee via a standard application procedure. Further details can be found at http://www.nshd.mrc.ac.uk/data. doi: 10.5522/NSHD/Q101; doi: 10.5522/NSHD/Q102; 10.5522/NSHD/Q103.

For peer review only

For peer review only - http://bmjopen.bmj.com/site/about/guidelines.xhtml

REFERENCES

1. Rice AS, Smith BH, Blyth FM. Pain and the global burden of disease. *Pain* 2016;157(4):791-6.

2. Thomas E. Pain in older people. In: Croft P, Blyth FM, van Der Windt D, eds. Chronic Pain Epidemiology: From Aetiology to Public Health. Oxford: OUP 2010:185-99.

3. Wolfe F, Smythe HA, Yunus MB, et al. The American College of Rheumatology 1990 Criteria for the Classification of Fibromyalgia. Report of the Multicenter Criteria Committee. *Arthritis Rheum* 1990;33(2):160-72.

4. Fayaz A, Croft P, Langford RM, et al. Prevalence of chronic pain in the UK: a systematic review and meta-analysis of population studies. *BMJ Open* 2016;6(6).

5. Mansfield KE, Sim J, Jordan JL, et al. A systematic review and meta-analysis of the prevalence of chronic widespread pain in the general population. *Pain* 2016;157(1):55-64.

6. Andrews P, Steultjens M, Riskowski J. Chronic widespread pain prevalence in the general population: A systematic review. *Eur J Pain* 2018;22(1):5-18.

7. Blyth FM, March LM, Brnabic AJ, et al. Chronic pain in Australia: a prevalence study. *Pain* 2001;89(2-3):127-34.

8. Gatchel RJ, Peng YB, Peters ML, et al. The biopsychosocial approach to chronic pain: scientific advances and future directions. *Psychol Bull* 2007;133(4):581.

9. Blyth FM, March LM, Brnabic AJ, et al. Chronic pain in Australia: a prevalence study. *Pain* 2001;89(2-3):127-34.

10. Bridges S. Chapter 9 Chronic Pain. In: Craig R, Mindell J, eds. Health Survey for England 2011. Leeds: Health and Social Care Information Centre 2011.

For peer review only - http://bmjopen.bmj.com/site/about/guidelines.xhtml

11. Elliott AM, Smith BH, Penny KI, et al. The epidemiology of chronic pain in the community. *Lancet* 1999;354(9186):1248-52.

12. Hagen K, Zwart JA, Svebak S, et al. Low socioeconomic status is associated with chronic musculoskeletal complaints among 46,901 adults in Norway. *Scand J Public Health* 2005;33(4):268-75.

13. Raftery MN, Sarma K, Murphy AW, et al. Chronic pain in the Republic of Ireland--community prevalence, psychosocial profile and predictors of pain-related disability: results from the Prevalence, Impact and Cost of Chronic Pain (PRIME) study, part 1. *Pain* 2011;152(5):1096-103.

14. Toblin RL, Mack KA, Perveen G, et al. A population-based survey of chronic pain and its treatment with prescription drugs. *Pain* 2011;152(6):1249-55.

15. Galobardes B, Lynch J, Smith GD. Measuring socioeconomic position in health research. *Br Med Bull* 2007;81-82:21-37.

16. Galobardes B, Shaw M, Lawlor DA, et al. Indicators of socioeconomic position (part 1). *J Epidemiol Comm Health* 2006;60(1):7-12.

17. Galobardes B, Shaw M, Lawlor DA, et al. Indicators of socioeconomic position (part 2). *J Epidemiol Comm Health* 2006;60(2):95-101.

18. Macfarlane GJ, Norrie G, Atherton K, et al. The influence of socioeconomic status on the reporting of regional and widespread musculoskeletal pain: results from the 1958 British Birth Cohort Study. *Ann Rheum Dis* 2009;68(10):1591-5.

19. Power C, Atherton K, Strachan DP, et al. Life-course influences on health in British adults: effects of socio-economic position in childhood and adulthood. *Int J Epidemiol* 2007;36(3):532-39.

20. Goosby BJ. Early life course pathways of adult depression and chronic pain. *J Health Soc Behav* 2013;54(1):75-91.

For peer review only - http://bmjopen.bmj.com/site/about/guidelines.xhtml

21. Ben-Shlomo Y, Kuh D. A life course approach to chronic disease epidemiology: conceptual models, empirical challenges and interdisciplinary perspectives. *Int J Epidemiol* 2002;31(2):285-93.

22. Wadsworth M, Kuh D, Richards M, et al. Cohort Profile: The 1946 National Birth Cohort (MRC National Survey of Health and Development). *Int J Epidemiol* 2006;35(1):49-54.

23. Wadsworth ME, Butterworth SL, Hardy RJ, et al. The life course prospective design: an example of benefits and problems associated with study longevity. *Soc Sci Med* 2003;57(11):2193-205.

24. Stafford M, Black S, Shah I, et al. Using a birth cohort to study ageing: representativeness and response rates in the National Survey of Health and Development. *Eur J Ageing* 2013;10(2):145-57.

25. Kuh D, Pierce M, Adams J, et al. Cohort profile: updating the cohort profile for the MRC National Survey of Health and Development: a new clinic-based data collection for ageing research. *Int J Epidemiol* 2011;40(1):e1-9.

26. Kuh D, Wong A, Shah I, et al. The MRC National Survey of Health and Development reaches age 70: maintaining participation at older ages in a birth cohort study. *Eur J Epidemiol* 2016;31(11):1135-47.

27. Muthuri SG, Kuh D, Bendayan R, et al. Chronic physical illness in early life and risk of chronic widespread and regional pain at age 68: evidence from the 1946 British birth cohort. *Pain* 2016;157(10):2382-9.

28. Heim N, Snijder MB, Deeg DJ, et al. Obesity in older adults is associated with an increased prevalence and incidence of pain. *Obesity* 2008;16(11):2510-7.

For peer review only - http://bmjopen.bmj.com/site/about/guidelines.xhtml

29. Mundal I, Grawe RW, Bjorngaard JH, et al. Prevalence and long-term predictors of persistent chronic widespread pain in the general population in an 11-year prospective study: the HUNT study. *BMC Musculoskelet Disord* 2014;15:213.

30. Hartvigsen J, Christensen K. Active lifestyle protects against incident low back pain in seniors: a population-based 2-year prospective study of 1387 Danish twins aged 70-100 years. *Spine* 2007;32(1):76-81.

31. Elliott AM, Smith BH, Hannaford PC, et al. The course of chronic pain in the community: results of a 4-year follow-up study. *Pain* 2002;99(1-2):299-307.

32. Goldberg DP, Hillier VF. A scaled version of the General Health Questionnaire. *Psychol Med* 1979;9(1):139-45.

33. van Buuren S, Groothuis-Oudshoorn K. mice: Multivariate Imputation by Chained Equations in R. *J Stat Soft* 2011;45(3):67.

34. Sterne JAC, White IR, Carlin JB, et al. Multiple imputation for missing data in epidemiological and clinical research: potential and pitfalls. *BMJ* 2009;338:b2393.

35. R: A language and environment for statistical computing [program]. Vienna, Austria: R Foundation for Statistical Computing, 2015.

36. RStudio: Integrated Development for R [program]. Boston, MA, USA: RStudio, Inc, 2015.

37. Venables WN , Ripley BD. Modern Applied Statistics with S. New York: Springer, 2002.

38. McMunn A, Lacey R, Worts D, et al. De-standardization and gender convergence in work–family life courses in Great Britain: A multi-channel sequence analysis. *Adv Life Course Res* 2015;26:60-75.

39. Stafford M, Lacey R, Murray E, et al. Work–family life course patterns and work participation in later life. *Eur J Ageing* 2018 doi: 10.1007/s10433-018-0470-7.

For peer review only

For peer review only - http://bmjopen.bmj.com/site/about/guidelines.xhtml

40. Woods V. Work-related musculoskeletal health and social support. *Occupational Med (Oxford)* 2005;55(3):177-89.

41. Christensen JO, Knardahl S. Work and neck pain: a prospective study of psychological, social, and mechanical risk factors. *Pain* 2010;151(1):162-73.

42. Kuh D, Hardy R, Langenberg C, et al. Mortality in adults aged 26-54 years related to socioeconomic conditions in childhood and adulthood: post war birth cohort study. *BMJ* 2002;325(7372):1076-80.

For peer review only

For peer review only - http://bmjopen.bmj.com/site/about/guidelines.xhtml

Table 1. Indicators of socioeconomic position used in analyses

Indicator	Definition	Age ascertained (ages used if missing at main age)
Childhood		
Paternal occupational class	Registrar General's Social Classification:<br>I & II – Professional and Managerial & technical<br>IIINM – Skilled non-manual<br>IIIM – Skilled manual<br>IV & V – Partly-skilled & Unskilled	4 (or 11 (n = 48) or 15 (n = 17))<br>Total = 2,258
Paternal and maternal education	Higher<br>Secondary only<br>Primary / less	6 (Paternal = 2,117; Maternal = 2,099)
Household overcrowding	> 2 persons per room in the household	4 (11 (n = 34) 15 (n = 13))<br>Total = 2,298
Housing tenure	Owner-occupier vs<br>Tenant (includes private and public tenants)	4 (or 11 (n = 42) or 15 (n = 12))<br>Total = 2,300
Lack of household amenities	Lacks access to hot running water, own bathroom or own kitchen (score indicating lacks 0 to all 3).	2 (or 11 (n = 157) or 15 (n = 11))<br>Total = 2,326
Earlier adulthood		
Own occupational class	Registrar General's Social Classification (categorised as above)	53 (or 43 (n = 123) or 36 (n = 59))<br>Total = 2,219
Home ownership	Owner-occupier vs<br>Tenant (includes private and public tenants)	53 (or 43 (n = 142) or 36 (n = 29))<br>Total = 2,294
Highest educational level attained	Degree or higher<br>A level or equivalent or less	26 (n = 2,297)
Financial hardship	None (no hardship)<br>Minimal (hard to manage but does not report going without necessities or unable to pay bills)<br>Moderate (hard to manage and either went without necessities or unable to pay bills)<br>Most (hard to manage, went without necessities and unable to pay bills)	43 (n = 2,150)
Later adulthood		
Home ownership	As in earlier adulthood	60-64 (n = 2,038)
Financial hardship	As in earlier adulthood	60-64 (n = 1,892)

For peer review only - http://bmjopen.bmj.com/site/about/guidelines.xhtml

Table 2. Characteristics of participants from of the MRC National Survey of Health and Development included in analyses (sample restricted to those with data on pain at age 68 and at least one indicator of SEP (maximum n=2,378))

		Women (n = 1,140)	Men (n = 1,238)
Pain at age 68			
	CWP	164 (13.3%)	89 (7.8%)
	CRP	400 (32.3%)	327 (28.7%)
	Other pain	155 (12.5%)	173 (15.2%)
	No pain	519 (41.9%)	551 (48.3%)
BMI at age 60-64; n = 1,906			
	$kg \cdot m^{-2}$ mean (SD)	27.8 (5.2)	27.8 (4.0)
Smoking at age 68, n = 2,356			
	Current	96 (7.8%)	112 (9.9%)
	Ex-smoker	653 (53.2%)	680 (60.2%)
	Never	478 (39.0%)	337 (29.9%)
Alcohol units at age 68, n = 2,135		2.0 (0.0, 6.0)	7.0 (2.0, 15.0)
Leisure time physical activity at age 68, n = 2,352			
	Inactive	733 (59.7%)	682 (60.7%)
	1 to 4 times / month	163 (13.3%)	120 (10.6%)
	5 or more times / month	330 (26.9%)	324 (28.7%)
Symptoms of anxiety and depression (GHQ-28) at age 60-64, n = 1,884			
	Yes (≤4)	222 (22.4%)	115 (12.9%)
	No (>4)	768 (77.6%)	779 (87.1%)
Marital status at age 68, n = 2,333			
	Single	39 (3.2%)	47 (4.2%)
	Married	856 (70.7%)	912 (81.2%)
	Separated / divorced	167 (13.8%)	109 (9.7%)
	Widowed	148 (12.2%)	55 (4.9%)
Childhood SEP (age 0 – 15)			
Father's occupational class	Class IV to V	291 (24.8%)	260 (24.0%)
Home ownership	Tenant status	826 (69.0%)	773 (70.1%)
Lack of amenities	Lacks three amenities	48 (4.0%)	47 (4.2%)
Overcrowding	Overcrowded	67 (5.6%)	39 (3.5%)
Maternal education	Primary or less	817 (74.4%)	742 (72.8%)
Paternal education	Primary or less	740 (67.8%)	667 (65.9%)
Earlier adulthood SEP (age 26 – 53)			
Educational level	A level or equivalent or less	1,116 (93.5%)	913 (82.8%)
Financial hardship	Most hardship	31 (2.8%)	23 (2.3%)
Occupational class	Class IV to V	193 (17.0%)	93 (8.6%)
Home ownership	Tenant status	128 (10.7%)	116 (10.6%)
Later adulthood SEP (age 60/64)			
Home ownership	Tenant status	283 (26.1%)	307 (32.2%)
Financial hardship	Most hardship	11 (1.1%)	19 (2.1%)

For brevity descriptive statistics for only the most adverse category of each SEP indicator are presented, a complete breakdown of the distribution of each SEP indicator by sex is given in Supplementary Tables 1 and 2. Numbers on each covariate vary due to missing data. CWP Chronic widespread pain; CRP Chronic regional pain; GHQ-28 General Health Questionnaire – 28 item; BMI Body Mass Index; SEP socioeconomic position.

For peer review only - http://bmjopen.bmj.com/site/about/guidelines.xhtml

Table 3. Associations between indicators of socioeconomic position in childhood and pain at age 68.

Exposure	n	Model	Exposure level	CWP v no pain RRR (95% CI)	CRP v no pain RRR (95% CI)	Other pain vs no pain RRR (95% CI)	p
Father's occupational class (ref = I-II)	2,258	1	IIINM	1.11 (0.73, 1.69)	0.94 (0.71, 1.26)	0.90 (0.62, 1.31)	.87
			IIIM	1.06 (0.71, 1.57)	1.09 (0.84, 1.41)	0.87 (0.62, 1.24)	
			IV – V	1.29 (0.87, 1.92)	1.00 (0.76, 1.32)	1.00 (0.70, 1.42)	
		2	IIINM	1.05 (0.68, 1.62)	0.93 (0.70, 1.24)	0.90 (0.62, 1.32)	.97
			IIIM	0.86 (0.57, 1.29)	1.01 (0.77, 1.31)	0.83 (0.58, 1.19)	
			IV – V	1.05 (0.69, 1.59)	0.93 (0.70, 1.23)	0.98 (0.68, 1.41)	
Home ownership (ref = owner-occupier)	2,300	1	Tenant	1.06 (0.78, 1.43)	1.05 (0.85, 1.29)	1.02 (0.77, 1.34)	.96
		2	Tenant	0.88 (0.64, 1.21)	0.97 (0.78, 1.20)	0.98 (0.74, 1.30)	.88
Lack of amenities (ref = lacks 0)	2,326	1	Lacks 1 or more	1.21 (0.92, 1.60)	1.03 (0.85, 1.24)	0.99 (0.77, 1.27)	.57
		2	Lacks 1 or more	1.14 (0.85, 1.52)	1.01 (0.83, 1.22)	0.98 (0.76, 1.26)	.81
Overcrowding (ref = not overcrowded)	2,298	1	Overcrowded	1.12 (0.59, 2.11)	1.17 (0.75, 1.82)	0.72 (0.36, 1.44)	.56
		2	Overcrowded	1.01 (0.52, 1.95)	1.13 (0.72, 1.77)	0.71 (0.35, 1.44)	.64
Maternal education (ref = diploma / degree)	2,117	1	Secondary	1.06 (0.58, 1.95)	0.76 (0.51, 1.13)	0.56 (0.34, 0.95)	.40
			Primary	1.08 (0.65, 1.82)	0.82 (0.59, 1.13)	0.75 (0.49, 1.13)	
		2	Secondary	1.01 (0.54, 1.88)	0.74 (0.50, 1.11)	0.57 (0.34, 0.97)	.38
			Primary	0.94 (0.55, 1.61)	0.76 (0.54, 1.06)	0.73 (0.48, 1.11)	
Paternal education (ref = diploma / degree)	2,099	1	Secondary	1.12 (0.66, 1.87)	1.26 (0.89, 1.78)	0.83 (0.53, 1.30)	.68
			Primary	1.11 (0.73, 1.67)	1.07 (0.81, 1.42)	0.84 (0.60, 1.20)	
		2	Secondary	0.99 (0.58, 1.69)	1.19 (0.84, 1.70)	0.80 (0.51, 1.27)	.64
			Primary	0.90 (0.58, 1.39)	0.97 (0.72, 1.30)	0.81 (0.57, 1.16)	

Model 1: Sex-adjusted; Model 2: adjusted for sex, BMI, smoking, alcohol, leisure-time physical activity, GHQ-28 caseness and marital status. CWP Chronic widespread pain; CRP Chronic regional pain.

For peer review only - http://bmjopen.bmj.com/site/about/guidelines.xhtml

Table 4. Associations between indicators of socioeconomic position in earlier and later adulthood and pain at age 68.

Exposure	n	Model	Exposure level	CWP v no pain<br>RRR (95% CI)	CRP v no pain<br>RRR (95% CI)	Other pain vs no pain<br>RRR (95% CI)	p
Earlier adulthood							
Educational level (women)* (ref = degree)	1,194	2	A level or equivalent or less	0.94 (0.44, 2.01)	1.93 (1.01, 3.69)	0.54 (0.29, 1.00)	<.01
Educational level (men)* (ref = degree)	1,103	2	A level or equivalent or less	2.68 (1.12, 6.45)	1.07 (0.74, 1.56)	1.45 (0.88, 2.40)	.06
Financial hardship (ref = none)	2,150	1	Minimal	1.37 (0.78, 2.42)	1.13 (0.75, 1.70)	0.98 (0.56, 1.71)	<.01
			Moderate	2.61 (1.38, 4.95)	1.31 (0.76, 2.28)	1.31 (0.64, 2.67)	
			Most	5.38 (2.56, 11.30)	1.81 (0.89, 3.72)	1.67 (0.67, 4.18)	
		2	Minimal	1.21 (0.67, 2.17)	1.09 (0.72, 1.65)	0.95 (0.54, 1.68)	.03
			Moderate	2.32 (1.19, 4.52)	1.32 (0.75, 2.30)	1.34 (0.65, 2.74)	
			Most	4.44 (2.02, 9.77)	1.79 (0.86, 3.74)	1.75 (0.69, 4.47)	
Occupational class (ref = I-II)	2,219	1	IIINM	1.15 (0.79, 1.66)	0.95 (0.73, 1.23)	1.16 (0.83, 1.61)	.15
			IIIM	1.41 (0.92, 2.19)	1.35 (1.02, 1.79)	0.97 (0.66, 1.41)	
			IV – V	1.52 (0.99, 2.34)	1.24 (0.91, 1.69)	0.94 (0.61, 1.45)	
		2	IIINM	1.08 (0.74, 1.59)	0.91 (0.70, 1.19)	1.13 (0.81, 1.58)	.38
			IIIM	1.29 (0.82, 2.03)	1.31 (0.98, 1.75)	0.93 (0.63, 1.38)	
			IV – V	1.22 (0.77, 1.92)	1.17 (0.85, 1.61)	0.93 (0.59, 1.44)	
Home ownership (ref = owner-occupier)	2,294	1	Tenant	1.81 (1.21, 2.72)	1.07 (0.77, 1.48)	1.33 (0.89, 1.98)	.03
		2	Tenant	1.62 (1.06, 2.49)	1.06 (0.76, 1.48)	1.36 (0.90, 2.05)	.11
Later adulthood							
Home ownership (ref = owner)	2,038	1	Tenant	1.25 (0.90, 1.73)	1.04 (0.83, 1.30)	0.90 (0.67, 1.22)	.44
		2	Tenant	1.07 (0.76, 1.51)	0.98 (0.78, 1.24)	0.89 (0.66, 1.21)	.83
Financial hardship (ref = none)	1,892	1	Minimal	1.61 (0.62, 4.16)	0.88 (0.40, 1.94)	0.77 (0.26, 2.30)	.52
			Moderate	2.03 (0.86, 4.80)	1.73 (0.90, 3.34)	1.54 (0.66, 3.62)	
			Most	2.26 (0.77, 6.64)	1.53 (0.66, 3.57)	0.89 (0.24, 3.20)	
		2	Minimal	1.39 (0.51, 3.74)	0.83 (0.37, 1.87)	0.77 (0.25, 2.34)	.89
			Moderate	1.47 (0.60, 3.63)	1.56 (0.79, 3.05)	1.49 (0.63, 3.54)	
			Most	1.71 (0.55, 5.29)	1.39 (0.58, 3.32)	0.91 (0.25, 3.34)	

Model 1: Sex-adjusted; Model 2: adjusted for sex, BMI, smoking, alcohol, leisure-time physical activity, GHQ-28 caseness and marital status.* The models for education are stratified by sex due to a significant interaction term between sex and education (Likelihood ratio test, $p < 0.001$). CWP Chronic widespread pain; CRP Chronic regional pain.

For peer review only - http://bmjopen.bmj.com/site/about/guidelines.xhtml

For peer review only

Table 5. Associations between cumulative exposure to tenant status and financial hardship and pain at age 68.

Exposure	n	Model	Accumulation	CWP vs no pain	CRP vs no pain RRR (95% CI)	Other pain vs no pain RRR (95% CI)	p
Housing ownership accumulation (ref = always owner)	1,956	1	Tenant at one point	0.90 (0.61, 1.33)	0.91 (0.70, 1.18)	0.89 (0.63, 1.25)	.43
			Tenant at two points	1.10 (0.68, 1.79)	1.08 (0.78, 1.50)	0.96 (0.62, 1.47)	
			Tenant at all points	1.99 (1.10, 3.59)	1.13 (0.71, 1.81)	1.17 (0.64, 2.11)	
		2	Tenant at one point	0.83 (0.55, 1.23)	0.85 (0.65, 1.11)	0.86 (0.61, 1.21)	.72
			Tenant at two points	0.90 (0.54, 1.49)	0.99 (0.71, 1.39)	0.92 (0.59, 1.43)	
			Tenant at all points	1.48 (0.78, 2.78)	0.99 (0.61, 1.60)	1.12 (0.60, 2.08)	
Financial hardship accumulation (ref = No or minimal hardship)	1,795	1	Hardship at one point	2.26 (1.33, 3.83)	1.36 (0.89, 2.08)	1.57 (0.93, 2.65)	<.01
			Hardship at both points	5.18 (1.70, 15.77)	1.74 (0.58, 5.20)	1.13 (0.23, 5.66)	
		2	Hardship at one point	1.93 (1.11, 3.35)	1.34 (0.86, 2.08)	1.59 (0.93, 2.73)	.09
			Hardship at both points	3.90 (1.20, 12.64)	1.60 (0.52, 4.92)	1.15 (0.23, 5.84)	

Model 1: Sex-adjusted; Model 2: adjusted for sex, BMI, smoking, alcohol, leisure-time physical activity, GHQ-28 caseness and marital status. CWP Chronic widespread pain; CRP Chronic regional pain.

For peer review only

For peer review only - http://bmjopen.bmj.com/site/about/guidelines.xhtml

Supplementary Table 1. Distribution of indicators of socioeconomic position in childhood by sex.

		Women n (%)	Men n (%)	Total n (%)
Father's occupational class	I-II	308 (26.3)	282 (26.0)	590 (26.1)
	IIINM	242 (20.6)	224 (20.7)	466 (20.6)
	IIIM	332 (28.3)	319 (39.4)	651 (28.8)
	IV-V	291 (24.8)	260 (24.0)	551 (24.4)
	Unknown	65	55	120
Home ownership	Owner-occupier	371 (31.0)	330 (29.9)	701 (30.5)
	Tenant	826 (69.0)	773 (70.1)	1599 (69.5)
	Unknown	41	37	78
Lack of amenities	0	629 (51.9)	597 (53.5)	1226 (52.7)
	1	205 (16.9)	192 (17.2)	397 (17.1)
	2	329 (27.2)	279 (25.0)	608 (26.1)
	3	48 (4.0)	47 (4.2)	95 (4.1)
	Unknown	27	25	52
Overcrowding	Not overcrowded	1128 (94.4)	1064 (96.5)	2192 (95.4)
	Overcrowded	67 (5.6)	39 (3.5)	106 (4.6)
	Unknown	43	37	80
Maternal education	Degree	39 (3.6)	45 (4.4)	84 (4.0)
	Diploma	80 (7.3)	57 (5.6)	137 (6.5)
	Secondary	162 (14.8)	175 (17.2)	337 (15.9)
	Primary	817 (74.4)	742 (72.8)	1,559 (73.6)
	Unknown	140	121	261
Paternal education	Degree	110 (10.1)	102 (10.1)	212 (10.1)
	Diploma	68 (6.2)	58 (5.8)	126 (6.0)
	Secondary	173 (15.9)	184 (18.3)	357 (17.0)
	Primary	740 (67.8)	667 (65.9)	1,404 (66.9)
	Unknown	147	132	279

For peer review only

Supplementary Table 2. Distribution of indicators of socioeconomic position in earlier adulthood by sex.

		Women n (%)	Men n (%)	Total n (%)
Educational level (age 26)	Degree or higher	78 (6.5)	190 (17.2)	268 (11.7)
	A level or equivalent or less	1,116 (93.5)	913 (82.8)	2,029 (88.3)
	Unknown	44	37	81
Hard to manage financially (age 43)*	No	977 (78.9)	911 (88.7)	1888 (87.5)
	Yes	154 (12.4)	116 (11.3)	270 (12.5)
	Unknown	107	113	220
Go without necessities (age 43)*	No	1023 (90.1)	943 (91.6)	1966 (90.8)
	Yes	113 (10.0)	87 (8.5)	200 (9.2)
	Unknown	102	110	212
Unable to pay bills (Age 43)*	No	1082 (95.4)	968 (94.4)	2,050 (94.9)
	Yes	52 (4.6)	58 (5.7)	110 (5.1)
	Unknown	105	115	220
Derived financial hardship (age 43)	None	974 (86.4)	906 (88.7)	1,880 (87.4)
	Minimal	75 (6.7)	61 (6.0)	136 (6.3)
	Moderate	48 (4.3)	32 (3.1)	80 (3.7)
	Most	31 (2.8)	23 (2.3)	54 (2.5)
	Unknown	110	118	228
Occupational class (age 53)	I-II	437 (38.5)	619 (57.1)	1056 (47.6)
	IIINM	418 (36.9)	109 (10.1)	527 (23.8)
	IIIM	86 (7.6)	264 (24.3)	350 (15.8)
	IV-V	193 (17.0)	93 (8.6)	286 (12.9)
	Unknown	104	55	159
Home ownership (age 53)	Owner-occupier	1071 (89.3)	979 (89.4)	2055 (89.4)
	Tenant	128 (10.7)	116 (10.6)	244 (10.6)
	Unknown	40	45	85

* These three variables were used to derive the financial hardship variable.

For peer review only

Supplementary Table 3. Distribution of indicators of socioeconomic position in later adulthood by sex.

		Women n (%)	Men n (%)	Total n (%)
Home ownership	Owner-occupier	802 (73.9)	646 (67.8)	1,448 (71.1)
	Tenant	283 (26.1)	307 (32.2)	590 (29.0)
	Unknown	187	153	340
Hard to manage financially*	No	945 (94.1)	828 (93.1)	1773 (93.7)
	Yes	59 (5.9)	61 (6.9)	120 (6.3)
	Unknown	234	251	485
Go without necessities*	No	911 (90.7)	794 (89.1)	1705 (90.0)
	Yes	93 (9.3)	97 (10.9)	190 (10.0)
	Unknown	234	249	483
Unable to pay bills*	No	974 (97.1)	854 (75.9)	1,828 (96.5)
	Yes	29 (2.9)	37 (4.2)	66 (3.5)
	Unknown	235	249	485
Derived financial hardship	None	944 (94.1)	828 (93.1)	1,772 (93.7)
	Minimal	18 (1.8)	19 (2.1)	37 (2.0)
	Moderate	30 (3.0)	23 (2.6)	53 (2.8)
	Most	11 (1.1)	19 (2.1)	30 (1.6)
	Unknown	237	252	489

* These three variables were used to derive the financial hardship variable.

For peer review only

Supplementary Table 4. Associations between indicators of socioeconomic position in childhood and pain at age 68.

Exposure	n	Model	Exposure level	CWP vs no pain RRR (95% CI)	CRP vs no pain RRR (95% CI)	Other pain vs no pain RRR (95% CI)	p
Father's occupational class (ref = I-II)	2258	1	IIINM	1.11 (0.73, 1.68)	0.94 (0.71, 1.25)	0.90 (0.62, 1.31)	.86
			IIIM	1.05 (0.71, 1.55)	1.09 (0.84, 1.41)	0.87 (0.62, 1.24)	
			IV – V	1.30 (0.87, 1.92)	1.00 (0.76, 1.32)	1.00 (0.70, 1.42)	
		2	IIINM	1.11 (0.73, 1.69)	0.94 (0.71, 1.26)	0.90 (0.62, 1.31)	.87
			IIIM	1.06 (0.71, 1.57)	1.09 (0.84, 1.41)	0.87 (0.62, 1.24)	
			IV – V	1.29 (0.87, 1.92)	1.00 (0.76, 1.32)	1.00 (0.70, 1.42)	
		3	IIINM	1.10 (0.72, 1.67)	0.94 (0.70, 1.25)	0.90 (0.62, 1.31)	.91
			IIIM	0.90 (0.60, 1.34)	1.02 (0.78, 1.33)	0.83 (0.58, 1.18)	
			IV – V	1.07 (0.71, 1.61)	0.94 (0.71, 1.24)	0.96 (0.67, 1.38)	
		4	IIINM	1.10 (0.72, 1.69)	0.93 (0.70, 1.25)	0.90 (0.62, 1.31)	.89
			IIIM	1.02 (0.69, 1.52)	1.07 (0.83, 1.39)	0.87 (0.61, 1.23)	
			IV – V	1.26 (0.84, 1.88)	0.99 (0.75, 1.30)	0.99 (0.70, 1.41)	
		5	IIINM	1.05 (0.68, 1.62)	0.93 (0.70, 1.24)	0.90 (0.62, 1.32)	.91
			IIIM	0.86 (0.57, 1.29)	1.01 (0.77, 1.31)	0.83 (0.58, 1.19)	
			IV – V	1.05 (0.69, 1.59)	0.93 (0.70, 1.23)	0.98 (0.68, 1.41)	
Home ownership (ref = owner)	2300	1	Tenant	1.05 (0.77, 1.42)	1.05 (0.85, 1.29)	1.02 (0.77, 1.34)	.97
		2	Tenant	1.06 (0.78, 1.43)	1.05 (0.85, 1.29)	1.02 (0.77, 1.34)	.96
		3	Tenant	0.92 (0.67, 1.26)	0.98 (0.80, 1.22)	0.98 (0.74, 1.30)	.96
		4	Tenant	1.01 (0.75, 1.38)	1.03 (0.84, 1.27)	.01 (0.77, 1.33)	.99
		5	Tenant	0.88 (0.64, 1.21)	0.97 (0.78, 1.20)	0.98 (0.74, 1.30)	.88
Lack of amenities (ref = lacks 0)	2326	1	Lacks 1 or more	1.22 (0.93, 1.62)	1.03 (0.85, 1.25)	0.99 (0.77, 1.27)	.53
		2	Lacks 1 or more	1.21 (0.92, 1.60)	1.03 (0.85, 1.24)	0.99 (0.77, 1.27)	.57
		3	Lacks 1 or more	1.12 (0.84, 1.48)	0.99 (0.82, 1.21)	0.97 (0.75, 1.25)	.85
		4	Lacks 1 or more	1.24 (0.94, 1.65)	1.04 (0.86, 1.26)	0.99 (0.77, 1.27)	.48
		5	Lacks 1 or more	1.14 (0.85, 1.52)	1.01 (0.83, 1.22)	0.98 (0.76, 1.26)	.81
Overcrowding (ref = not overcrowded)	2298	1	Overcrowded	1.20 (0.64, 2.27)	1.20 (0.77, 1.87)	0.72 (0.36, 1.44)	.47
		2	Overcrowded	1.12 (0.59, 2.11)	1.17 (0.75, 1.82)	0.72 (0.36, 1.44)	.56
		3	Overcrowded	1.06 (0.56, 2.03)	1.15 (0.73, 1.80)	0.70 (0.35, 1.42)	.58
		4	Overcrowded	1.06 (0.56, 2.03)	1.14 (0.73, 1.79)	0.71 (0.35, 1.43)	.60
		5	Overcrowded	1.01 (0.52, 1.95)	1.13 (0.72, 1.77)	0.71 (0.35, 1.44)	.64
Maternal education (ref = dip / degree)	2117	1	Secondary	1.02 (0.56, 1.87)	0.74 (0.50, 1.11)	0.57 (0.34, 0.96)	.42
			Primary	1.07 (0.64, 1.79)	0.81 (0.58, 1.13)	0.75 (0.50, 1.13)	
		2	Secondary	1.06 (0.58, 1.95)	0.76 (0.51, 1.13)	0.56 (0.34, 0.95)	.40
			Primary	1.08 (0.65, 1.82)	0.82 (0.59, 1.13)	0.75 (0.49, 1.13)	
		3	Secondary	1.04 (0.56, 1.91)	0.74 (0.50, 1.10)	0.57 (0.34, 0.96)	.34
			Primary	0.94 (0.55, 1.58)	0.75 (0.53, 1.04)	0.72 (0.48, 1.10)	

For peer review only

		4	Secondary	1.07 (0.58, 1.97)	0.76 (0.51, 1.13)	0.57 (0.34, 0.95)	.40
			Primary	1.11 (0.66, 1.88)	0.82 (0.59, 1.15)	0.75 (0.50, 1.13)	
		5	Secondary	1.01 (0.54, 1.88)	0.74 (0.50, 1.11)	0.57 (0.34, 0.97)	.38
			Primary	0.94 (0.55, 1.61)	0.76 (0.54, 1.06)	0.73 (0.48, 1.11)	
Paternal Education (ref = dip / degree)	2099	1	Secondary	1.08 (0.64, 1.81)	1.24 (0.87, 1.76)	0.83 (0.53, 1.30)	.71
			Primary	1.10 (0.73, 1.66)	1.07 (0.81, 1.42)	0.84 (0.60, 1.20)	
		2	Secondary	1.12 (0.66, 1.87)	1.26 (0.89, 1.78)	0.83 (0.53, 1.30)	.68
			Primary	1.11 (0.73, 1.67)	1.07 (0.81, 1.42)	0.84 (0.60, 1.20)	
		3	Secondary	1.03 (0.61, 1.75)	1.21 (0.85, 1.72)	0.81 (0.51, 1.27)	.60
			Primary	0.89 (0.58, 1.37)	0.97 (0.73, 1.30)	0.80 (0.56, 1.15)	
		4	Secondary	1.09 (0.64, 1.84)	1.24 (0.87, 1.77)	0.82 (0.52, 1.30)	.70
			Primary	1.09 (0.72, 1.66)	1.06 (0.80, 1.41)	0.84 (0.59, 1.19)	
		5	Secondary	0.99 (0.58, 1.69)	1.19 (0.84, 1.70)	0.80 (0.51, 1.27)	.64
			Primary	0.90 (0.58, 1.39)	0.97 (0.72, 1.30)	0.81 (0.57, 1.16)	

1. Unadjusted; 2. Adjusted for sex; 3. Adjusted for sex, BMI, smoking, alcohol, exercise; 4. Adjusted for sex and GHQ-28 caseness; 5. Adjusted for sex, BMI, smoking, alcohol, leisure-time physical activity, GHQ-28 caseness and marital status

For peer review only

Supplementary Table 5. Associations between indicators of socioeconomic position in earlier adulthood and pain at age 68.

Exposure	n	Model	Exposure level	CWP vs no pain RRR (95% CI)	CRP vs no pain RRR (95% CI)	Other pain vs no pain RRR (95% CI)	p
Educational level* Women (ref = degree)	1194	1	A level or equivalent or less	1.10 (0.53, 2.27)	2.04 (1.08, 3.84)	0.54 (0.30, 0.98)	<.01
		3	A level or equivalent or less	0.85 (0.40, 1.79)	1.83 (0.96, 3.46)	0.52 (0.28, 0.96)	<.01
		4	A level or equivalent or less	1.22 (0.58, 2.57)	2.15 (1.14, 4.08)	0.56 (0.31, 1.03)	<.01
		5	A level or equivalent or less	0.94 (0.44, 2.01)	1.93 (1.01, 3.69)	0.54 (0.29, 1.00)	<.01
Educational level* Men (ref = degree)	1103	1	A level or equivalent or less	3.32 (1.41, 7.80)	1.11 (0.77, 1.59)	1.51 (0.93, 2.46)	<.01
		3	A level or equivalent or less	2.64 (1.11, 6.32)	1.06 (0.73, 1.53)	1.45 (0.88, 2.39)	.06
		4	A level or equivalent or less	3.35 (1.41, 7.93)	1.11 (0.78, 1.60)	1.51 (0.93, 2.47)	<.01
		5	A level or equivalent or less	2.68 (1.12, 6.45)	1.07 (0.74, 1.56)	1.45 (0.88, 2.40)	.06
Financial hardship (ref = none)	2150	1	Minimal	1.40 (0.80, 2.46)	1.14 (0.76, 1.71)	0.98 (0.56, 1.71)	<.01
			Moderate	2.73 (1.45, 5.15)	1.34 (0.78, 2.33)	1.31 (0.64, 2.67)	
			Most	5.46 (2.62, 11.39)	1.83 (0.89, 3.74)	1.67 (0.67, 4.18)	
		2	Minimal	1.37 (0.78, 2.42)	1.13 (0.75, 1.70)	0.98 (0.56, 1.71)	<.01
			Moderate	2.61 (1.38, 4.95)	1.31 (0.76, 2.28)	1.31 (0.64, 2.67)	
			Most	5.38 (2.56, 11.30)	1.81 (0.89, 3.72)	1.67 (0.67, 4.18)	
		3	Minimal	1.29 (0.72, 2.28)	1.09 (0.72, 1.64)	0.95 (0.54, 1.66)	<.01
			Moderate	2.49 (1.30, 4.76)	1.33 (0.76, 2.31)	1.32 (0.65, 2.70)	
			Most	4.85 (2.28, 10.35)	1.74 (0.84, 3.58)	1.68 (0.67, 4.23)	
		4	Minimal	1.30 (0.73, 2.30)	1.10 (0.73, 1.66)	0.97 (0.55, 1.69)	<.01
			Moderate	2.44 (1.27, 4.67)	1.27 (0.73, 2.21)	1.29 (0.63, 2.63)	
			Most	4.96 (2.33, 10.57)	1.75 (0.85, 3.60)	1.65 (0.66, 4.13)	
		5	Minimal	1.21 (0.67, 2.17)	1.09 (0.72, 1.65)	0.95 (0.54, 1.68)	.03
			Moderate	2.32 (1.19, 4.52)	1.32 (0.75, 2.30)	1.34 (0.65, 2.74)	
			Most	4.44 (2.02, 9.77)	1.79 (0.86, 3.74)	1.75 (0.69, 4.47)	
Occupational class (ref = I-IIINM)	2219	1	IIINM	1.46 (1.03, 2.09)	1.06 (0.83, 1.36)	1.13 (0.83, 1.54)	0.09
			IIIM	1.25 (0.81, 1.92)	1.28 (0.97, 1.69)	0.98 (0.67, 1.43)	
			IV – V	1.80 (1.17, 2.75)	1.34 (0.99, 1.82)	0.92 (0.60, 1.42)	
		2	IIINM	1.15 (0.79, 1.66)	0.95 (0.73, 1.23)	1.16 (0.83, 1.61)	.15
			IIIM	1.41 (0.92, 2.19)	1.35 (1.02, 1.79)	0.97 (0.66, 1.41)	
			IV – V	1.52 (0.99, 2.34)	1.24 (0.91, 1.69)	0.94 (0.61, 1.45)	
		3	IIINM	1.09 (0.75, 1.59)	0.93 (0.71, 1.20)	1.14 (0.82, 1.59)	.44
			IIIM	1.22 (0.78, 1.90)	1.29 (0.96, 1.71)	0.94 (0.64, 1.39)	
			IV – V	1.26 (0.80, 1.97)	1.19 (0.87, 1.64)	0.93 (0.60, 1.44)	
		4	IIINM	1.11 (0.76, 1.62)	0.93 (0.71, 1.21)	1.15 (0.83, 1.60)	.17
			IIIM	1.45 (0.93, 2.25)	1.36 (1.02, 1.81)	0.97 (0.66, 1.42)	
			IV – V	1.44 (0.93, 2.23)	1.20 (0.88, 1.65)	0.93 (0.60, 1.43)	

For peer review only

		5	IIINM	1.08 (0.74, 1.59)	0.91 (0.70, 1.19)	1.13 (0.81, 1.58)	.38
			IIIM	1.29 (0.82, 2.03)	1.31 (0.98, 1.75)	0.93 (0.63, 1.38)	
			IV – V	1.22 (0.77, 1.92)	1.17 (0.85, 1.61)	0.93 (0.59, 1.44)	
Home ownership (ref = owner)	2294	1	Tenant	1.81 (1.21, 2.70)	1.07 (0.77, 1.48)	1.33 (0.89, 1.98)	.03
		2	Tenant	1.81 (1.21, 2.72)	1.07 (0.77, 1.48)	1.33 (0.89, 1.98)	.03
		3	Tenant	1.60 (1.06, 2.42)	1.02 (0.73, 1.41)	1.33 (0.89, 1.99)	.10
		4	Tenant	1.79 (1.19, 2.69)	1.06 (0.77, 1.47)	1.33 (0.89, 1.98)	.04
		5	Tenant	1.62 (1.06, 2.49)	1.06 (0.76, 1.48)	1.36 (0.90, 2.05)	.11

1. Unadjusted; 2. Adjusted for sex; 3. Adjusted for sex, BMI, smoking, alcohol and leisure-time physical activity; 4. Adjusted for sex and GHQ-28 caseness; 5. Adjusted for sex, BMI, smoking, alcohol, leisure-time physical activity, GHQ-28 caseness and marital status * The models for education are stratified by sex due to a significant interaction term ($p < 0.001$). Model 2 is therefore omitted and no adjustment for sex is made.

For peer review only

Supplementary Table 6. Associations between indicators of socioeconomic position in later adulthood and pain at age 68.

Exposure	n	Model	Exposure level	CWP vs no pain RRR (95% CI)	CRP v no pain RRR (95% CI)	Other pain vs no pain RRR (95% CI)	p
Home ownership (ref = owner)	2038	1	Tenant	1.17 (0.85, 1.62)	1.01 (0.81, 1.26)	0.90 (0.67, 1.22)	.62
		2	Tenant	1.25 (0.90, 1.73)	1.04 (0.83, 1.30)	0.90 (0.67, 1.22)	.44
		3	Tenant	1.08 (0.78, 1.52)	0.97 (0.77, 1.22)	0.88 (0.65, 1.19)	.74
		4	Tenant	1.20 (0.86, 1.67)	1.02 (0.81, 1.28)	0.90 (0.67, 1.21)	.55
		5	Tenant	1.07 (0.76, 1.51)	0.98 (0.78, 1.24)	0.89 (0.66, 1.21)	.83
Financial hardship (ref = none)	1892	1	Minimal	1.55 (0.60, 3.98)	0.87 (0.40, 1.92)	0.77 (0.26, 2.31)	.58
			Moderate	2.06 (0.88, 4.85)	1.74 (0.90, 3.35)	1.54 (0.66, 3.61)	
			Most	1.99 (0.68, 5.80)	1.48 (0.64, 3.43)	0.89 (0.25, 3.22)	
		2	Minimal	1.61 (0.62, 4.16)	0.88 (0.40, 1.94)	0.77 (0.26, 2.30)	.52
			Moderate	2.03 (0.86, 4.80)	1.73 (0.90, 3.34)	1.54 (0.66, 3.62)	
			Most	2.26 (0.77, 6.64)	1.53 (0.66, 3.57)	0.89 (0.24, 3.20)	
		3	Minimal	1.49 (0.57, 3.94)	0.84 (0.38, 1.86)	0.76 (0.25, 2.28)	.64
			Moderate	1.79 (0.74, 4.28)	1.64 (0.85, 3.18)	1.52 (0.64, 3.58)	
			Most	2.35 (0.79, 7.01)	1.51 (0.64, 3.53)	0.89 (0.24, 3.24)	
		4	Minimal	1.48 (0.56, 3.89)	0.84 (0.38, 1.86)	0.75 (0.25, 2.26)	.86
			Moderate	1.61 (0.67, 3.88)	1.54 (0.79, 2.99)	1.46 (0.62, 3.44)	
			Most	1.67 (0.55, 5.04)	1.31 (0.56, 3.09)	0.82 (0.23, 3.00)	
		5	Minimal	1.39 (0.51, 3.74)	0.83 (0.37, 1.87)	0.77 (0.25, 2.34)	.89
			Moderate	1.47 (0.60, 3.63)	1.56 (0.79, 3.05)	1.49 (0.63, 3.54)	
			Most	1.71 (0.55, 5.29)	1.39 (0.58, 3.32)	0.91 (0.25, 3.34)	

1. Unadjusted; 2. Adjusted for sex; 3. Adjusted for sex, BMI, smoking, alcohol and leisure-time physical activity; 4. Adjusted for sex and GHQ-28 caseness; 5. Adjusted for sex, BMI, smoking, alcohol, leisure-time physical activity, GHQ-28 caseness and marital status

For peer review only

Supplementary Table 7. Changes in SEP measures between early and later time periods.

	n (%)	
Tenant status (n = 1,956)	*Tenant status in later adulthood*	
Tenant status in childhood	Owner-occupier	Tenant
Owner-occupier	471 (23.8)	127 (6.4)
Tenant	931 (47.1)	446 (22.6)
Financial hardship (n = 1,795)	*Financial hardship later adulthood*	
Financial hardship earlier adulthood	No (none or minimal)	Yes (moderate or most)
No (none or minimal)	1637 (91.2)	52 (2.9)
Yes (moderate or most)	84 (4.7)	22 (1.2)

For peer review only

Supplementary Table 8. Accumulation of occupational class and financial hardship.

Accumulation	Tenant status	Financial hardship
	n (%)	
0 (high at both time points)	453 (23.2)	1,637 (91.2)
1	1017 (52.0)	136 (7.6)
2	352 (18.0)	22 (1.2)
3	134 (6.9)	-
Unknown	422	583

For peer review only

Supplementary Table 9. Associations between cumulative exposure to tenant status and financial hardship and pain at age 68.

Exposure	n	Model	Exposure level	CWP vs no pain RRR (95% CI)	CRP v no pain RRR (95% CI)	Other pain vs no pain RRR (95% CI)	p
Financial hardship accumulation (ref = No or minimal hardship)	1795	1	Hardship at one point	2.23 (1.32, 3.78)	1.36 (0.88, 2.08)	1.57 (0.93, 2.65)	<.01
			Hardship at both	5.44 (1.80, 16.39)	1.77 (0.59, 5.28)	1.14 (0.23, 5.68)	
		2	Hardship at one point	2.26 (1.33, 3.83)	1.36 (0.89, 2.08)	1.57 (0.93, 2.65)	<.01
			Hardship at both	5.18 (1.70, 15.77)	1.74 (0.58, 5.20)	1.13 (0.23, 5.66)	
		3	Hardship at one point	2.17 (1.27, 3.71)	1.35 (0.88, 2.08)	1.59 (0.93, 2.70)	.02
			Hardship at both	4.45 (1.42, 13.94)	1.63 (0.54, 4.92)	1.12 (0.22, 5.64)	
		4	Hardship at one point	1.99 (1.16, 3.41)	1.28 (0.83, 1.97)	1.53 (0.90, 2.59)	.05
			Hardship at both	4.26 (1.36, 13.32)	1.57 (0.52, 4.76)	1.09 (0.22, 5.45)	
		5	Hardship at one point	1.93 (1.11, 3.35)	1.34 (0.86, 2.08)	1.59 (0.93, 2.73)	.09
			Hardship at both	3.90 (1.20, 12.64)	1.60 (0.52, 4.92)	1.15 (0.23, 5.84)	
Housing ownership accumulation (ref = always owner)	1956	1	Tenant at one point	0.90 (0.61, 1.32)	0.91 (0.70, 1.18)	0.89 (0.63, 1.25)	.47
			Tenant at two points	1.02 (0.63, 1.65)	1.05 (0.76, 1.45)	0.95 (0.62, 1.47)	
			Tenant at all points	1.96 (1.09, 3.52)	1.13 (0.71, 1.80)	1.16 (0.64, 2.11)	
		2	Tenant at one point	0.90 (0.61, 1.33)	0.91 (0.70, 1.18)	0.89 (0.63, 1.25)	.43
			Tenant at two points	1.10 (0.68, 1.79)	1.08 (0.78, 1.50)	0.96 (0.62, 1.47)	
			Tenant at all points	1.99 (1.10, 3.59)	1.13 (0.71, 1.81)	1.17 (0.64, 2.11)	
		3	Tenant at one point	0.84 (0.56, 1.24)	0.86 (0.66, 1.12)	0.86 (0.61, 1.21)	.73
			Tenant at two points	0.93 (0.56, 1.53)	0.98 (0.70, 1.37)	0.90 (0.58, 1.39)	
			Tenant at all points	1.50 (0.81, 2.78)	0.97 (0.60, 1.57)	1.07 (0.58, 1.98)	
		4	Tenant at one point	0.89 (0.60, 1.32)	0.91 (0.70, 1.18)	0.89 (0.63, 1.25)	.60
			Tenant at two points	1.05 (0.64, 1.72)	1.06 (0.76, 1.47)	0.95 (0.62, 1.46)	
			Tenant at all points	1.83 (1.00, 3.34)	1.10 (0.69, 1.76)	1.15 (0.64, 2.09)	
		5	Tenant at one point	0.83 (0.55, 1.23)	0.85 (0.65, 1.11)	0.86 (0.61, 1.21)	.72
			Tenant at two points	0.90 (0.54, 1.49)	0.99 (0.71, 1.39)	0.92 (0.59, 1.43)	
			Tenant at all points	1.48 (0.78, 2.78)	0.99 (0.61, 1.60)	1.12 (0.60, 2.08)	

1. Unadjusted; 2. Adjusted for sex; 3. Adjusted for sex, BMI, smoking, alcohol and leisure-time physical activity; 4. Adjusted for sex and GHQ-28 caseness; 5. Adjusted for sex, BMI, smoking, alcohol, leisure-time physical activity, GHQ-28 caseness and marital status

For peer review only

STROBE 2007 (v4) checklist of items to be included in reports of observational studies in epidemiology*
Checklist for cohort, case-control, and cross-sectional studies (combined)

Section/Topic	Item #	Recommendation	Reported on page #
**Title and abstract**	1	(*a*) Indicate the study's design with a commonly used term in the title or the abstract	1
		(*b*) Provide in the abstract an informative and balanced summary of what was done and what was found	3
**Introduction**			
Background/rationale	2	Explain the scientific background and rationale for the investigation being reported	5-6
Objectives	3	State specific objectives, including any pre-specified hypotheses	6
**Methods**			
Study design	4	Present key elements of study design early in the paper	7
Setting	5	Describe the setting, locations, and relevant dates, including periods of recruitment, exposure, follow-up, and data collection	7-8
Participants	6	(*a*) *Cohort study*—Give the eligibility criteria, and the sources and methods of selection of participants. Describe methods of follow-up<br>*Case-control study*—Give the eligibility criteria, and the sources and methods of case ascertainment and control selection. Give the rationale for the choice of cases and controls<br>*Cross-sectional study*—Give the eligibility criteria, and the sources and methods of selection of participants	7
		(*b*) *Cohort study*—For matched studies, give matching criteria and number of exposed and unexposed<br>*Case-control study*—For matched studies, give matching criteria and the number of controls per case	N/A
Variables	7	Clearly define all outcomes, exposures, predictors, potential confounders, and effect modifiers. Give diagnostic criteria, if applicable	8-9 & Table 1
Data sources/ measurement	8*	For each variable of interest, give sources of data and details of methods of assessment (measurement). Describe comparability of assessment methods if there is more than one group	8-9
Bias	9	Describe any efforts to address potential sources of bias	10-11
Study size	10	Explain how the study size was arrived at	7
Quantitative variables	11	Explain how quantitative variables were handled in the analyses. If applicable, describe which groupings were chosen and why	10-11
Statistical methods	12	(*a*) Describe all statistical methods, including those used to control for confounding	10-11
		(*b*) Describe any methods used to examine subgroups and interactions	10-11
		(*c*) Explain how missing data were addressed	10-11
		(*d*) *Cohort study*—If applicable, explain how loss to follow-up was addressed<br>*Case-control study*—If applicable, explain how matching of cases and controls was addressed	N/A

		Cross-sectional study—If applicable, describe analytical methods taking account of sampling strategy	
		(*e*) Describe any sensitivity analyses	11
**Results**			
Participants	13*	(a) Report numbers of individuals at each stage of study—eg numbers potentially eligible, examined for eligibility, confirmed eligible, included in the study, completing follow-up, and analysed	12
		(b) Give reasons for non-participation at each stage	7
		(c) Consider use of a flow diagram	N/A
Descriptive data	14*	(a) Give characteristics of study participants (eg demographic, clinical, social) and information on exposures and potential confounders	12
		(b) Indicate number of participants with missing data for each variable of interest	10 & Table 2
		(c) *Cohort study*—Summarise follow-up time (eg, average and total amount)	7-9
Outcome data	15*	*Cohort study*—Report numbers of outcome events or summary measures over time	12 & Table 2
		Case-control study—Report numbers in each exposure category, or summary measures of exposure	N/A
		Cross-sectional study—Report numbers of outcome events or summary measures	N/A
Main results	16	(*a*) Give unadjusted estimates and, if applicable, confounder-adjusted estimates and their precision (eg, 95% confidence interval). Make clear which confounders were adjusted for and why they were included	12-13 & Tables 3 to 5
		(*b*) Report category boundaries when continuous variables were categorized	9 (GHQ-28 scores)
		(*c*) If relevant, consider translating estimates of relative risk into absolute risk for a meaningful time period	N/A
Other analyses	17	Report other analyses done—eg analyses of subgroups and interactions, and sensitivity analyses	12-14 & Tables 3 to 5
**Discussion**			
Key results	18	Summarise key results with reference to study objectives	15
Limitations	19	Discuss limitations of the study, taking into account sources of potential bias or imprecision. Discuss both direction and magnitude of any potential bias	18
Interpretation	20	Give a cautious overall interpretation of results considering objectives, limitations, multiplicity of analyses, results from similar studies, and other relevant evidence	19
Generalisability	21	Discuss the generalisability (external validity) of the study results	15-17
**Other information**			
Funding	22	Give the source of funding and the role of the funders for the present study and, if applicable, for the original study on which the present article is based	19

*Give information separately for cases and controls in case-control studies and, if applicable, for exposed and unexposed groups in cohort and cross-sectional studies.

Note: An Explanation and Elaboration article discusses each checklist item and gives methodological background and published examples of transparent reporting. The STROBE checklist is best used in conjunction with this article (freely available on the Web sites of PLoS Medicine at http://www.plosmedicine.org/, Annals of Internal Medicine at http://www.annals.org/, and Epidemiology at http://www.epidem.com/). Information on the STROBE Initiative is available at www.strobe-statement.org.

For peer review only
